# Neurological diagnoses in hospitalized COVID-19 patients associated with adverse outcomes: A multinational cohort study

**DOI:** 10.1371/journal.pdig.0000484

**Published:** 2024-04-15

**Authors:** Meghan R. Hutch, Jiyeon Son, Trang T. Le, Chuan Hong, Xuan Wang, Zahra Shakeri Hossein Abad, Michele Morris, Alba Gutiérrez-Sacristán, Jeffrey G. Klann, Anastasia Spiridou, Ashley Batugo, Riccardo Bellazzi, Vincent Benoit, Clara-Lea Bonzel, William A. Bryant, Lorenzo Chiudinelli, Kelly Cho, Priyam Das, Tomás González González, David A. Hanauer, Darren W. Henderson, Yuk-Lam Ho, Ne Hooi Will Loh, Adeline Makoudjou, Simran Makwana, Alberto Malovini, Bertrand Moal, Danielle L. Mowery, Antoine Neuraz, Malarkodi Jebathilagam Samayamuthu, Fernando J. Sanz Vidorreta, Emily R. Schriver, Petra Schubert, Jeffery Talbert, Amelia L. M. Tan, Byorn W. L. Tan, Bryce W. Q. Tan, Valentina Tibollo, Patric Tippman, Guillaume Verdy, William Yuan, Paul Avillach, Nils Gehlenborg, Gilbert S. Omenn, The Consortium for Clinical Characterization of COVID-19 by EHR (4CE), Shyam Visweswaran, Tianxi Cai, Yuan Luo, Zongqi Xia

**Affiliations:** 1 Department of Preventive Medicine, Northwestern University, Chicago, Illinois, United States of America; 2 Department of Neurology, University of Pittsburgh, Pittsburgh, Pennsylvania, United States of America; 3 Department of Biostatistics, Epidemiology, and Informatics, University of Pennsylvania Perelman School of Medicine, Philadelphia, Pennsylvania, United States of America; 4 Department of Biostatistics and Bioinformatics, Duke University, Durham, North Carolina, United States of America; 5 Department of Population Health Sciences, University of Utah, Salt Lake City, Utah, United States of America; 6 Department of Biomedical Informatics, Harvard Medical School, Boston, Massachusetts, United States of America; 7 Department of Biomedical Informatics, University of Pittsburgh, Pittsburgh, Pennsylvania, United States of America; 8 Department of Medicine, Massachusetts General Hospital, Boston, Massachusetts, United States of America; 9 Digital Research, Informatics and Virtual Environments (DRIVE), Great Ormond Street Hospital for Children, London, United Kingdom; 10 Department of Electrical, Computer and Biomedical Engineering, University of Pavia, Pavia, Italy; 11 IT Department, Innovation & Data, APHP Greater Paris University Hospital, Paris, France; 12 UOC Ricerca, Innovazione e Brand reputation, ASST Papa Giovanni XXIII, Bergamo, Italy; 13 Population Health and Data Science, VA Boston Healthcare System, Boston Massachusetts, United States of America; 14 Massachusetts Veterans Epidemiology Research and Information Center (MAVERIC), VA Boston Healthcare System, Boston Massachusetts, United States of America; 15 Health Informatics, Hospital Universitario 12 de Octubre, Madrid, Spain; 16 Department of Learning Health Sciences, University of Michigan Medical School, Ann Arbor, Michigan, United States of America; 17 Center for Clinical and Translational Science, University of Kentucky, Lexington, Kentucky, United States of America; 18 Department of Anaesthesia, National University Health System, Kent Ridge, Singapore; 19 Institute of Medical Biometry and Statistics, Faculty of Medicine and Medical Center, University of Freiburg, Freiburg, Germany; 20 Laboratory of Informatics and Systems Engineering for Clinical Research, Istituti Clinici Scientifici Maugeri SpA SB IRCCS, Pavia, Italy; 21 IAM Unit, Bordeaux University Hospital, Bordeaux, France; 22 Department of biomedical informatics, Hôpital Necker-Enfants Malade, Assistance Publique Hôpitaux de Paris (APHP), University of Paris, Paris, France; 23 Department of Medicine, David Geffen School of Medicine at UCLA, Los Angeles, California, United States of America; 24 Data Analytics Center, University of Pennsylvania Health System, Philadelphia, Pennsylvania, United States of America; 25 Division of Biomedical Informatics, University of Kentucky, Lexington, Kentucky, United States of America; 26 Department of Medicine, National University Hospital, Singapore, Kent Ridge, Singapore; 27 Institute of Medical Biometry and University of Freiburg, Medical Center, Freiburg, Germany; 28 Departments of Computational Medicine & Bioinformatics, Internal Medicine, Human Genetics, Public Health, University of Michigan, Ann Arbor, Michigan, United States of America; University of Florida, UNITED STATES

## Abstract

Few studies examining the patient outcomes of concurrent neurological manifestations during acute COVID-19 leveraged multinational cohorts of adults and children or distinguished between central and peripheral nervous system (CNS vs. PNS) involvement. Using a federated multinational network in which local clinicians and informatics experts curated the electronic health records data, we evaluated the risk of prolonged hospitalization and mortality in hospitalized COVID-19 patients from 21 healthcare systems across 7 countries. For adults, we used a federated learning approach whereby we ran Cox proportional hazard models locally at each healthcare system and performed a meta-analysis on the aggregated results to estimate the overall risk of adverse outcomes across our geographically diverse populations. For children, we reported descriptive statistics separately due to their low frequency of neurological involvement and poor outcomes. Among the 106,229 hospitalized COVID-19 patients (104,031 patients ≥18 years; 2,198 patients <18 years, January 2020-October 2021), 15,101 (14%) had at least one CNS diagnosis, while 2,788 (3%) had at least one PNS diagnosis. After controlling for demographics and pre-existing conditions, adults with CNS involvement had longer hospital stay (11 versus 6 days) and greater risk of (Hazard Ratio = 1.78) and faster time to death (12 versus 24 days) than patients with no neurological condition (NNC) during acute COVID-19 hospitalization. Adults with PNS involvement also had longer hospital stay but lower risk of mortality than the NNC group. Although children had a low frequency of neurological involvement during COVID-19 hospitalization, a substantially higher proportion of children with CNS involvement died compared to those with NNC (6% vs 1%). Overall, patients with concurrent CNS manifestation during acute COVID-19 hospitalization faced greater risks for adverse clinical outcomes than patients without any neurological diagnosis. Our global informatics framework using a federated approach (versus a centralized data collection approach) has utility for clinical discovery beyond COVID-19.

## Author summary

Patients hospitalized with acute COVID-19 may concurrently develop central (CNS) or peripheral (PNS) nervous system manifestations that worsen clinical outcomes. Few studies have explicitly distinguished between CNS and PNS involvement when evaluating risks of adverse outcomes. We examined how CNS versus PNS involvement during acute COVID-19 affects patient outcomes by developing a methodological framework to harness the diverse multi-national health records for clinical discovery while preserving patient confidentiality. We applied federated learning methods and rigorous local data quality control to study over 100,000 adults and children hospitalized with acute COVID-19 from 21 healthcare systems spanning 7 countries while ensuring that individual-level patient data remain at the respective healthcare system. We found that adults with CNS or PNS involvement had longer hospitalizations than patients without neurologic involvement. Adults with CNS involvement had an increased risk of death, and the frequency of death was likewise higher in children with CNS involvement. This large study of a geographically diverse cohort of adult and pediatric patients highlights the need for vigilance and prompt treatment of acute COVID-19 patients with CNS involvement. Our global informatics framework using a federated approach (versus a centralized data collection approach) has utility beyond clinical investigation pertaining to COVID-19.

## Introduction

After exposure to the severe acute respiratory syndrome coronavirus 2 (SARS-CoV-2), both adults and children experience a wide range of acute neurological manifestations. Following the start of the pandemic, early case series [1–5] and small cohort studies from single healthcare systems [6–9] reported the occurrence of acute neurological manifestations in adults with Coronavirus-19 (COVID-19). Neurological diagnoses during acute COVID-19 such as ischemic stroke [10], intracranial hemorrhage [3], seizures [4], or meningoencephalitis [5] were associated with adverse clinical outcomes in adults, including higher rates of in-hospital mortality [6,9,11] and lower rates of home discharge [6]. Additionally, altered mental status and stroke significantly increased in-hospital mortality regardless of disease severity [9]. Similar findings have since been confirmed by larger multicentered studies [12–14]. Taken together, these studies highlight the importance of early recognition and treatment of concurrent neurological manifestations during acute COVID-19.

While less common than adults, prior case series and small multicentered studies documented rare life-threatening neurological manifestations in children with acute COVID-19, including severe encephalopathy, infectious encephalitis, acute disseminated encephalomyelitis, and ischemic or hemorrhagic stroke [15–19]. In one multinational study examining acute COVID-19 hospitalization, adults and children were found to have different neurological manifestations and outcomes, though the study did not stratify the analysis by more granular age groups (*e*.*g*., younger vs. older children) and focused primarily on comparing critically ill patients versus otherwise [20]. Thus, there is a need to validate these findings in additional cohorts of adults and children.

We previously leveraged the scalable, federated, multinational network of the Consortium for Clinical Characterization of COVID-19 by Electronic Health Records (EHR) [21–23] (4CE; www.covidclinical.net) to assess the incidence of neurological diagnoses in hospitalized acute COVID-19 patients [24,25]. Central to the 4CE federated approach and distinguishing from other COVID-19 research using EHR data is the critical data quality control performed by *local* clinician and informatics experts following the adoption of a standardized data collection approach by each healthcare system. Our approach enables large-scale, multinational study design without compromising data quality or patient confidentiality. In this study, we aimed to evaluate the risk of adverse clinical outcomes in hospitalized adults and children with acute COVID-19 and concurrent neurological manifestations across age groups using the 4CE network.

For all hospitalized patients with acute COVID-19, we categorized neurological status based on involvement of the central nervous system (CNS) versus peripheral nervous system (PNS), which differ in pathophysiology, diagnosis, treatment, and prognosis. We assessed adult and pediatric patients separately. In the larger adult population, we performed covariate-adjusted survival analyses to assess the risk of prolonged hospitalization or mortality in patients who experienced concurrent neurological diagnoses during acute COVID-19 hospitalization when compared to those who did not. In the smaller pediatric population, we presented descriptive statistics with respect to adverse clinical outcomes. Finally, to our knowledge, we have curated and made available one of the largest multinational datasets containing the aggregated counts, proportions, and clinical trajectories of hospitalized COVID-19 adults and children with and without concurrent CNS and PNS diagnoses.

## Methods

### Ethics statement

Each participating healthcare system obtained Institutional Review Board (IRB) approval from local governing ethics committee, each with an approval of a waiver of informed consent for both adults and children, because access of de-identified data and external sharing of summary statistics (without interaction with the patients) are deemed minimal risk.

IRB Approval was obtained at Assistance Publique—Hôpitaux de Paris, Boston Children’s Hospital, Bordeaux University Hospital, Great Ormond Street Hospital for Children, ASST Papa Giovanni XXIII Bergamo, Istituti Clinici Scientifici Maugeri, Hospital Universitario 12 de Octubre, Madrid, Spain, Massachusetts General Brigham, National University Hospital, Northwestern University, Medical Center at University of Freiburg, University of Kentucky, University of Pittsburgh/UPMC, VA North Atlantic, VA Southwest, VA Midwest, VA Continental, and VA Pacific. An exempt determination was made by the IRB at University of California Los Angeles, University of Michigan, and University of Pennsylvania.

### Patients and data

In March 2020, the 4CE consortium began assembling EHR data from hospitalized patients with positive SARS-CoV-2 reverse transcription-polymerase chain reaction (RT-PCR) tests. Following consortium guidelines, participating healthcare systems collected patient-level clinical data, including demographics, medical history, admission, and discharge dates. Data underwent standardized quality control checks by local clinicians and informatics experts. In this study, we analyzed COVID-19 hospitalization data from January 2020 to October 2021 from 21 healthcare systems, encompassing 293 hospitals, across 7 countries. October 2021 was chosen as a cutoff date to focus on the pre-Omicron periods and to reduce the potential confounding effects of vaccine boosters on the analysis. Each participating healthcare system (S1 Table) received local Institutional Review Board approval. We provided details on healthcare system-specific measures to protect patient confidentiality in the Supporting Information (S1 Methods).

COVID-19 hospitalization was defined as the first hospital admission that occurred between 7 days before and up to 14 days after the first positive SARS-CoV-2 PCR test. For all hospitalized COVID-19 patients, we collected International Classification of Diseases (ICD)-10 and/or ICD-9 codes depending on the healthcare system, as previously described [24]. The dataset included pre-admission diagnoses defined by all ICD codes that occurred -365 to -15 days before the first (index) COVID-19 hospital admission date as well as post-admission diagnoses defined by ICD codes that occurred anytime on or after the index admission date. Following the pre-planned consortium-wide guideline, we excluded ICD codes within 14 days preceding the index admission date as a conservative approach to mitigate inflation or uncertainty regarding whether these codes might represent early symptoms or signs of COVID-19.

To address potential variability in coding practices across diverse multinational healthcare systems, diagnoses were represented by the major (or parent) ICD category codes (first three alphanumeric characters before the decimal point). As a part of the initial 4CE guideline, the use of the parent ICD category codes harmonized broader diagnosis capture despite documentation and practice differences among the participating healthcare systems. The parent ICD category codes also helped reduce statistical testing burden by grouping patients with similar diagnoses (under the same parent category) that may have similar clinical presentations and pathology.

### Neurological status during COVID-19 hospitalization

To classify patient-level neurological status during the first acute COVID-19 hospitalization, we assessed the occurrence of 21 ICD-10 (and 29 corresponding ICD-9) codes representing neurological diagnoses during acute COVID-19. Two neurologists (JS, ZX) compiled the list at study inception after reviewing the existing COVID-19 literature up to that point. We categorized neurological diagnosis codes as either CNS or PNS (S2 Table). We designated a patient’s neurological status during the first COVID-19 hospitalization as “CNS” or “PNS” if they had at least one ICD code in the respective category. A patient without a consensus neurological diagnosis code during the first COVID-19 hospitalization was designated as “NNC” for having no neurological condition. We excluded patients with both CNS and PNS diagnoses during acute COVID-19 hospitalization (<2% of the total patients) from the analysis due to potential confounding of exposures.

### Covariates

#### Pre-existing health condition or comorbidity estimation

We used the *icd* package [26] to map each patient’s prior diagnosis codes (-365 to -15 days preceding the index admission date for COVID-19 hospitalization) to the 29 pre-admission health conditions or comorbidities comprising the Elixhauser Comorbidity Index (ECI) [27,28] (S3–S4 Tables). Each comorbidity of the ECI was used as an individual covariate in our downstream survival models to adjust for a patient’s pre-existing comorbidity burden. We also calculated a weighted score for each patient’s overall pre-admission comorbidity burden (Table 1) using Van Walraven weights (S3 Table) [29].

**Table 1:** Study Population Characteristics.

	Entire Cohort	NNC ^1^	CNS ^1^	PNS ^1^	P-value ^2^
**All Patients, N (% of cohort)**	106229 (100%)	88340 (83.1%)	15101 (14.2%)	2788 (2.6%)	
**Sex, N (%)**					< .001*
Female	37590 (35.4%)	32399 (36.7%)	4247 (28.1%)	944 (33.9%)	
Male	68638 (64.6%)	55940 (63.3%)	10854 (71.9%)	1844 (66.1%)	
Unknown Sex	1 (0%)	1 (0%)	0 (0%)	0 (0%)	
**Age, N (%)**					
0–2	769 (0.7%)	747 (0.8%)	22 (0.1%)	0 (0%)	< .001*
3–5	275 (0.3%)	251 (0.3%)	24 (0.2%)	0 (0%)	< .001*
6–11	403 (0.4%)	368 (0.4%)	33 (0.2%)	2 (0.1%)	< .001*
12–17	707 (0.7%)	637 (0.7%)	60 (0.4%)	10 (0.4%)	< .001*
18–25	2328 (2.2%)	2138 (2.4%)	145 (1%)	45 (1.6%)	< .001*
26–49	17799 (16.8%)	16122 (18.3%)	1166 (7.7%)	511 (18.5%)	< .001*
50–69	36885 (34.7%)	31556 (35.7%)	4169 (27.7%)	1160 (41.9%)	< .001*
70–79	24860 (23.4%)	19672 (22.3%)	4508 (30%)	680 (24.6%)	< .001*
80+	22094 (20.8%)	16813 (19%)	4920 (32.7%)	361 (13%)	< .001*
**Race, N (%)**					
American Indian	350 (0.3%)	295 (0.3%)	55 (0.4%)	0 (0%)	.01*
Asian	1694 (1.6%)	1464 (1.7%)	205 (1.4%)	25 (0.9%)	< .001*
Black	16815 (15.8%)	13368 (15.1%)	2995 (19.9%)	452 (16.5%)	< .001*
Hawaiian / Pacific Islander	297 (0.3%)	255 (0.3%)	41 (0.3%)	1 (0%)	.054
Hispanic / Latino	870 (0.8%)	819 (0.9%)	29 (0.2%)	22 (0.8%)	< .001*
White	41871 (39.4%)	33360 (37.8%)	7401 (49.1%)	1110 (40.5%)	< .001*
Other / Not Recorded	44220 (41.6%)	38750 (43.9%)	4336 (28.8%)	1134 (41.3%)	< .001*
**Pre-admission Elixhauser score, Mean (SD)** ^**3**^	0.3 (0.7)	0.2 (0.5)	1.4 (2)	0.6 (1.7)	.014*
**Pre-admission Conditions (Selected), N (%)** ^**4**^					
Hypertension	35587 (33.5%)	27323 (30.9%)	7277 (48.2%)	987 (35.4%)	< .001*
Alcohol abuse	30130 (28.4%)	23065 (26.1%)	6194 (41%)	871 (31.2%)	< .001*
Drug abuse	26596 (25%)	20287 (23%)	5528 (36.6%)	781 (28%)	< .001*
Diabetes	21969 (20.7%)	16905 (19.1%)	4429 (29.3%)	635 (22.8%)	< .001*
**Number of pre-admission CNS codes, Mean (SD)** ^**3**^	0 (0)	0 (0)	0 (0)	0 (0)	.192
**Number of pre-admission PNS codes, Mean (SD)** ^**3**^	0 (0)	0 (0)	0 (0)	0 (0.1)	.062
**Severe Status, N (%)**					< .001*
Non-Severe	66252 (62.4%)	57771 (65.4%)	7007 (46.4%)	1474 (52.7%)	
Severe	39985 (37.6%)	30576 (34.6%)	8084 (53.6%)	1325 (47.3%)	
**Time to severe status, Mean days (SD)** ^**3**^	0.6 (1)	0.8 (1.3)	0.4 (0.7)	0.8 (1.1)	.503
**Survival, N (%)**					< .001*
Alive	87376 (82.3%)	74389 (84.2%)	10392 (68.8%)	2595 (93.1%)	
Deceased	18849 (17.7%)	13953 (15.8%)	4705 (31.2%)	191 (6.9%)	
**Time to death, Mean days (SD)** ^**3**^	15.3 (6.4)	15.9 (5.8)	22.4 (32.5)	36.9 (41.4)	.54
**Time to discharge, Mean days (SD)** ^**3**^	8.4 (4.2)	6.5 (3.6)	12.8 (6.1)	14.5 (18.3)	< .001*
**Readmission, N (%)**					
Not Readmitted	91646 (86.3%)	76877 (87%)	12435 (82.4%)	2334 (83.4%)	< .001*
Readmitted	14569 (13.7%)	11456 (13%)	2650 (17.6%)	463 (16.6%)	
**Time to first readmission, Mean days (SD)** ^**3**^	28.1 (20.3)	26.4 (17.6)	31.9 (33.7)	38.1 (28.4)	.504
**Number of readmissions, Mean (SD)** ^**3**^	0.1 (0.3)	0 (0)	0.1 (0.3)	0.1 (0.3)	.098

Notes

1. Neurological status during acute COVID-19 hospitalization: NNC = No Neurological Condition; CNS = Central Nervous System diagnosis; PNS = Peripheral Nervous System diagnosis.

2. P-values were adjusted with the Benjamini-Hochberg method to control the false discovery rate when evaluating the distribution (categorical variables) or means (continuous variables) of characteristics stratified by neurological status. P-values < .05 were deemed significant and designated with asterisk (*).

3. Continuous variables reflect the overall cohort mean and standard deviation (SD) of the median values reported by each healthcare system.

4. We report the top four most frequent pre-admission health conditions (*i*.*e*., comorbidities) in the adult population. Please see S7 Table for all pre-admission health conditions stratified by neurological status during acute COVID-19 hospitalization for both adult and pediatric populations.

5. Please see S9 Table and S10 Table for separate demographic tables as stratified by adult and pediatric populations, respectively.

#### Prior neurological conditions

To better account for each patient’s pre-existing neurological burden (*i*.*e*., neurological conditions not otherwise captured by the ECI), we first counted the total number of occurrences (z) of pre-admission neurological diagnosis codes for each patient in the period (-365 to -15 days) preceding the index admission date for COVID-19 hospitalization. We curated pre-existing CNS and PNS diagnoses (S2 Table) as two separate covariates for downstream survival analysis by transforming the total number of code occurrences (z) for a patient as log(z+1) to normalize the data.

#### Other covariates

Downstream survival models were also adjusted for age group, sex, and race/ethnicity (Table 1). Following 4CE guidelines, age was categorized into groups (0–2 years old, 3–5, 6–11, 12–17, 18–25, 26–49, 50–69, 70–79, 80+, “unknown” if not recorded). Race and ethnicity were considered as one variable and categorized as American Indian, Asian, Black, Hawaiian/Pacific Islander, Hispanic/Latino, White, and Other/Not recorded. Race and ethnicity are not available in certain countries outside of the United States (US).

### Primary outcomes

The primary endpoints were the time to two clinically important endpoints: hospital discharge and death. Patients met the endpoint if the event occurred within 90 days of the first COVID-19 hospitalization or were otherwise censored. We chose 90 days to capture events during the acute phase rather than the post-acute or long COVID phase, consistent with the World Health Organization guideline [30]. For the mortality outcome, we considered all-cause mortality due to the difficulty of distinguishing death directly due to COVID-19 versus a neurological complication [31].

Due to the low frequency of pediatric patients in our cohort with pre-existing health conditions and adverse clinical outcomes during acute COVID-19 hospitalization, we included only adults ≥18 years in the survival and comorbidity analysis. We assessed the descriptive statistics of the pediatric patients as stratified by neurological status during acute COVID-19 hospitalization.

### Primary statistical analyses

#### Population characteristics

We stratified the study population by neurological status to evaluate baseline demographics, pre-existing burden of neurological and other health conditions (*i*.*e*., comorbidity burden), and clinical characteristics during the acute COVID-19 hospitalization (Table 1). For each continuous variable, we report the overall mean and standard deviation (SD) of the median value recorded at each healthcare system. Categorical variables were assessed using chi-square tests, while continuous variables were evaluated using a Kruskal-Wallis one-way ANOVA. All p-values were adjusted with the Benjamini-Hochberg method to control the false discovery rate.

#### Local healthcare system-specific survival analysis of adult patients

**Fig 1:**
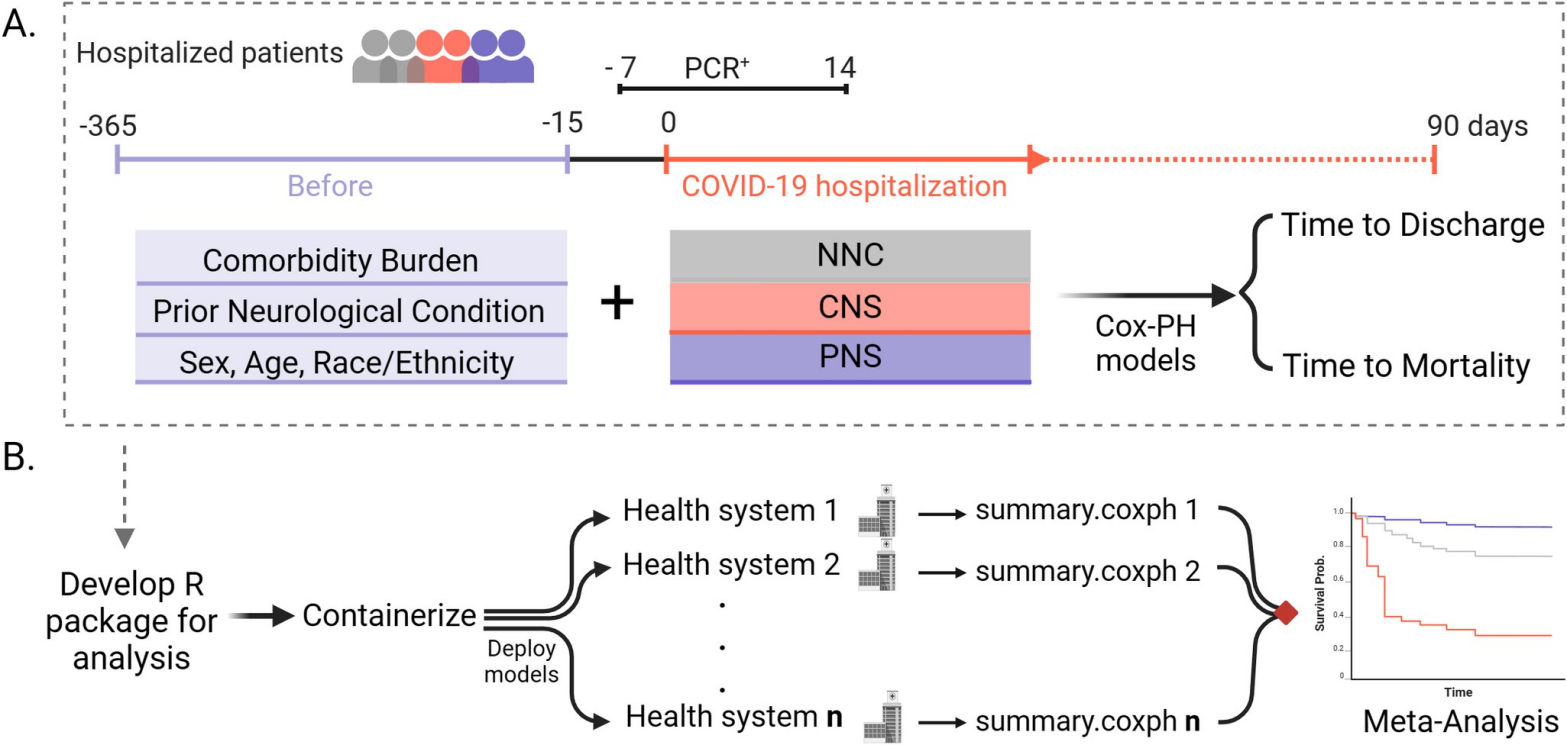
Study design and federated learning approach. **A.** We constructed Cox proportional hazard models to evaluate clinical endpoints in acute COVID-19 patients with concurrent neurological diagnoses. Patients were followed up to 90 days after the first acute COVID-19 hospital admission. Models were adjusted for pre-existing comorbidity burden and prior neurological conditions as well as baseline demographics, including age group, sex, race/ethnicity. **B.** The analysis plan was provided as a standardized R package and containerized with Docker to facilitate local deployment at each participating healthcare system. Cox proportional hazards statistics (summary.coxph) were extracted from the analysis at each healthcare system and included in a random-effects meta-analysis to pool the summary statistics. NNC: No Neurological Condition; CNS: Central Nervous System diagnosis; PNS: Peripheral Nervous System diagnosis.

At each healthcare system, we examined the association of concurrent neurological status during the first COVID-19 hospitalization with each clinical endpoint (in adults) using covariate-adjusted Cox proportional hazard models to evaluate time to hospital discharge and to death. We assessed the time to discharge *alive* using a competing risk model to control for death [32]. Importantly, we excluded patients who reached either clinical endpoint (discharge or death) on the first day of hospitalization. For each model, we controlled for pre-admission comorbidity burden by including each of the 29 health conditions of the ECI as individual covariates in the model, the number of pre-admission CNS and PNS diagnosis codes, and demographic variables (*i*.*e*., age group, sex, race/ethnicity). After each participating healthcare system deployed the patient-level analysis locally through a customized R (http://www.R-project.org) package deployed through Docker [33], we aggregated the summary statistics across all healthcare systems and performed a meta-analysis (Fig 1).

At each healthcare system, we estimated covariate-adjusted Kaplan-Meier time to event curves for each health outcome and neurological group. Specifically, for each outcome, we fit the Cox proportional hazards model to the patient cohort and estimated each patient’s survival rate, which is 1 minus event rate. Importantly, the survival rate was estimated for each patient by holding each patient’s covariates constant except for the neurological status. Thus, we estimated the survival rate for each neurological group, independent of the effect of additional covariates. Lastly, for each neurological group, we averaged all patients’ estimated survival rates to generate the overall survival curve.

#### Meta-analysis of hospitalized adults

We conducted a random-effects meta-analysis on locally generated results to compute the overall effect size of neurological status during acute COVID-19 hospitalization for each clinical endpoint. The use of the random-effects component controlled for the expected variation in patient population and clinical practice across healthcare systems. Separate random-effects models were constructed to compare the CNS group to the NNC group, and the PNS group to the NNC group. The generic inverse variance method was used to pool estimates across healthcare systems and the DerSimonian-Laird method was employed to estimate the between-health system variance (τ^2^) [34]. The global estimate of the association between neurological status during the first COVID-19 hospitalization and clinical endpoints was reported as hazard ratios (HR). Meta-analysis and forest plots were constructed using the meta [35] and forester [36] packages, respectively. Two healthcare systems with adult patients (NUH, UKFR) were excluded from the meta-analysis (but included for all other analyses) due to exceedingly low frequency of neurological diagnoses (<1% of adult patients). Healthcare systems with only pediatric patients were also excluded (BCH, GOSH) for the same reason. For the meta-analysis, results were deemed significant when p-values were below the Bonferroni-corrected threshold (p-value < .013 given four separate tests, Table 2).

When generating the pooled covariate-adjusted Kaplan-Meier survival curves for each clinical endpoint, we weighed each healthcare system by the average weights derived from both the CNS and PNS random-effects meta-analysis models, where the weight of each healthcare system k, was calculated as the inverse of the estimated variance (s^2^) + τ^2^, or:

$$w_{k}=\frac{1}{s^{2}+\tau^{2}}$$

### Secondary statistical analyses

#### Pre-existing health condition and risk of neurological diagnosis

To assess the contribution of pre-existing comorbidity on neurological status during acute COVID-19, we evaluated the relative risk of having a concurrent CNS or PNS diagnosis during the acute COVID-19 hospitalization for each of the pre-admission health conditions that constitute the ECl. The relative risk of having a CNS or PNS diagnosis was calculated for each pre-admission health condition by dividing the proportion of patients with the condition who developed a neurological diagnosis (CNS or PNS) during acute COVID-19 hospitalization, by the number of patients without the condition who developed a neurological diagnosis during COVID-19 hospitalization. We included all adult patients in this analysis.

#### Concurrence with COVID-19 severity

As a secondary outcome, we evaluated the risk of COVID-19 severity in our adult population. COVID-19 severity was defined as a binary variable based on a published EHR-based severity phenotype derived from diagnosis codes, laboratory orders, medication orders, and procedure codes that are proxies for respiratory distress and shock [37]. In our study, severe COVID-19 status also included death. Pediatric patients were excluded in this analysis due to the low incidence of neurological diagnoses and the lack of a validated severity phenotype in this population.

To evaluate the association between the occurrence of a neurological diagnosis and severe COVID-19 during acute COVID-19 hospitalization, we first fit a series of logistic regression models to predict the probability of a patient having the following events: a CNS diagnosis, a PNS diagnosis, severe COVID-19, CNS diagnosis ***and*** severe COVID-19, as well as PNS diagnosis ***and*** severe COVID-19. All models were adjusted for the same covariates used in the primary Cox-proportional hazards model.

Predicted probabilities were further evaluated using pointwise mutual information (PMI) to evaluate the association between the occurrence of a neurological diagnosis and severe COVID-19. Using CNS diagnosis and severe COVID-19 as an example, the joint probability of a patient having both CNS diagnosis and severe COVID-19 is divided by the product of the individual probabilities of a patient having CNS diagnosis and having severe COVID-19. With log(PMI(CNS,Severe)) > 0, a CNS diagnosis co-occurs with severe COVID-19 more frequently than expected under an independence assumption.


$$PMI(CNS,Severe)=log\left(\frac{p(CNS,Severe)}{p(CNS)*p(Severe)}\right)$$

The PMI(CNS, Severe) and PMI(PNS, Severe) were computed for each healthcare system individually. Confidence intervals were estimated with 500 bootstraps.

#### Model sensitivity analysis

We performed several sensitivity analyses to evaluate the performance of our Cox proportional hazard models. First, we constructed several models to compare the performance of three different comorbidity adjustment methods: 1) inclusion of the 29 comorbidities of the ECI as individual covariates, 2) the weighted ECI summary score as a single covariate, and 3) reducing the dimensionality of each healthcare system’s comorbidity matrix into the top 10 principal components [38,39] (S2 Methods). Additionally, we constructed models to evaluate the risk of adverse clinical outcomes within 30, 60, and 90 days after the index COVID-19 hospitalization admission date. We compared all models by evaluating the corresponding model concordance and hazard ratios (S4–S5 Figs). To further evaluate the age groups and the risk of adverse outcomes, we performed an additional random-effects meta-analysis using the locally estimated hazard ratios for each age group, which were used as covariates in the primary Cox-proportional hazards models.

### Code Availability

The R package used for deploying the analyses locally at each healthcare system is publicly available (https://github.com/covidclinical/Phase2.1NeuroRPackage/). The meta-analysis code and results are available in browsable and interactive R notebooks (https://github.com/covidclinical/Phase2.1NeuroAnalysis). All analyses were conducted using R (http://www.R-project.org) [40]. Fig 1 was created using Biorender (Biorender.com), while all other figures were created using ggplot2 [41].

## Results

### Patient characteristics

#### Demographics

**Fig 2:**
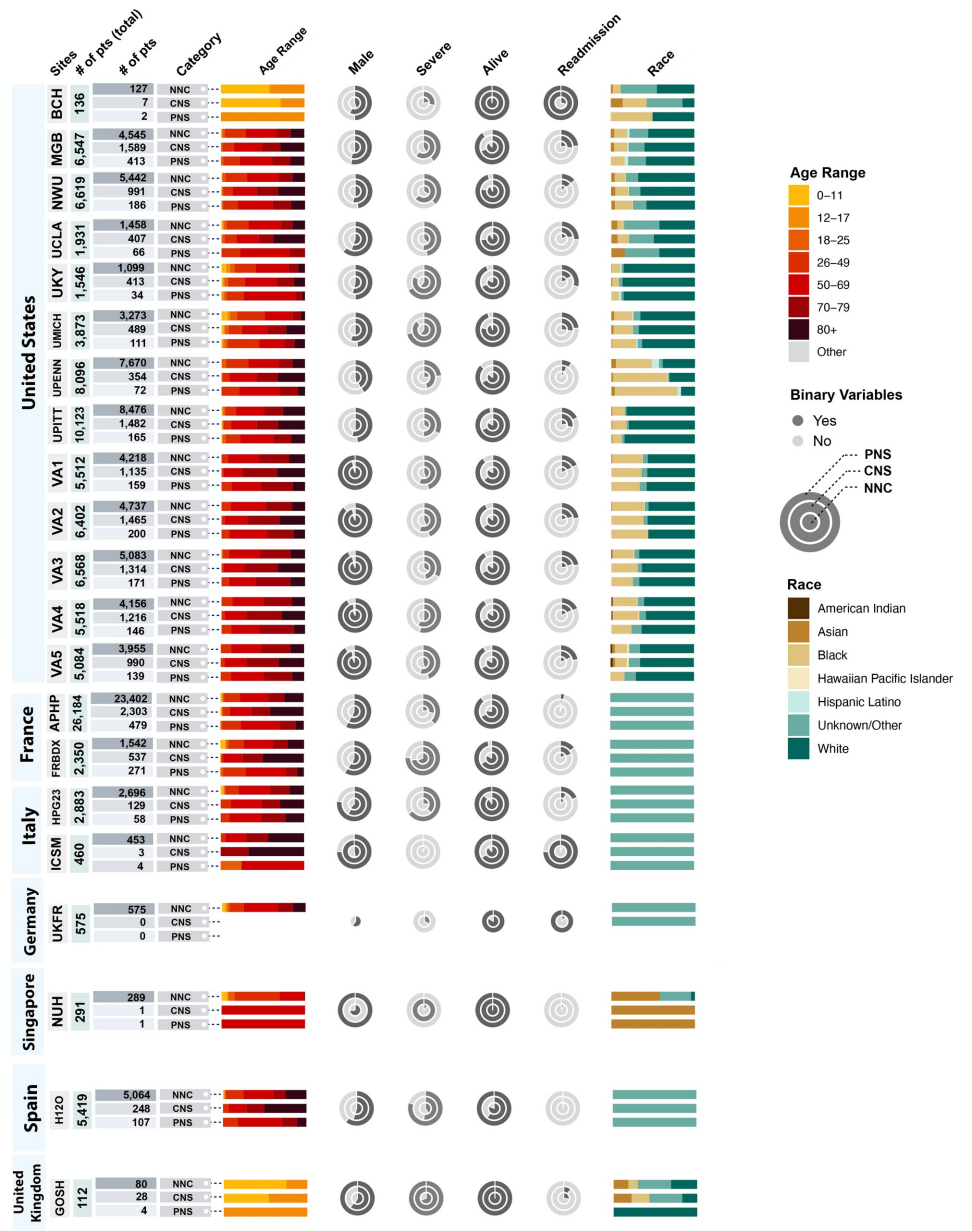
Demographic profile for each participating healthcare system arranged by country. Cohort-wise breakdown of the number of patients, age range, sex, severity status, mortality outcome, readmission status, and race at each healthcare system for each of the following neurological status during acute COVID-19 hospitalization: no neurological condition (NNC), central nervous system (CNS) diagnosis, and peripheral nervous system (PNS) diagnosis. Healthcare systems are arranged by country in descending order by the number of included participating healthcare systems. The stacked bar charts indicate the normalized distribution of age and race. The nested pie-charts are stratified by the neurological status with the darker portion representing the proportion of patients having the value of the binary variable for the given column header.

We analyzed data from 106,229 PCR-confirmed hospitalized patients with acute COVID-19 from 21 healthcare systems across 7 countries (Table 1, Fig 2, S1 Table). The US Veterans Affairs hospital system (comprising 170 hospitals) was divided into five regional healthcare systems, capturing broad geographic representations within the US. Males represented 65% of the overall study population. While 79% of the patients were 50 years or older, the study included 2,198 (2%) patients who were younger than 18 years of age. The study included 39% White, 16% Black, and 2% Asian as well as 42% “other/not recorded” since most non-US countries did not record race or ethnicity.

#### Frequency of neurological diagnosis during acute COVID-19 hospitalization

**Fig 3:**
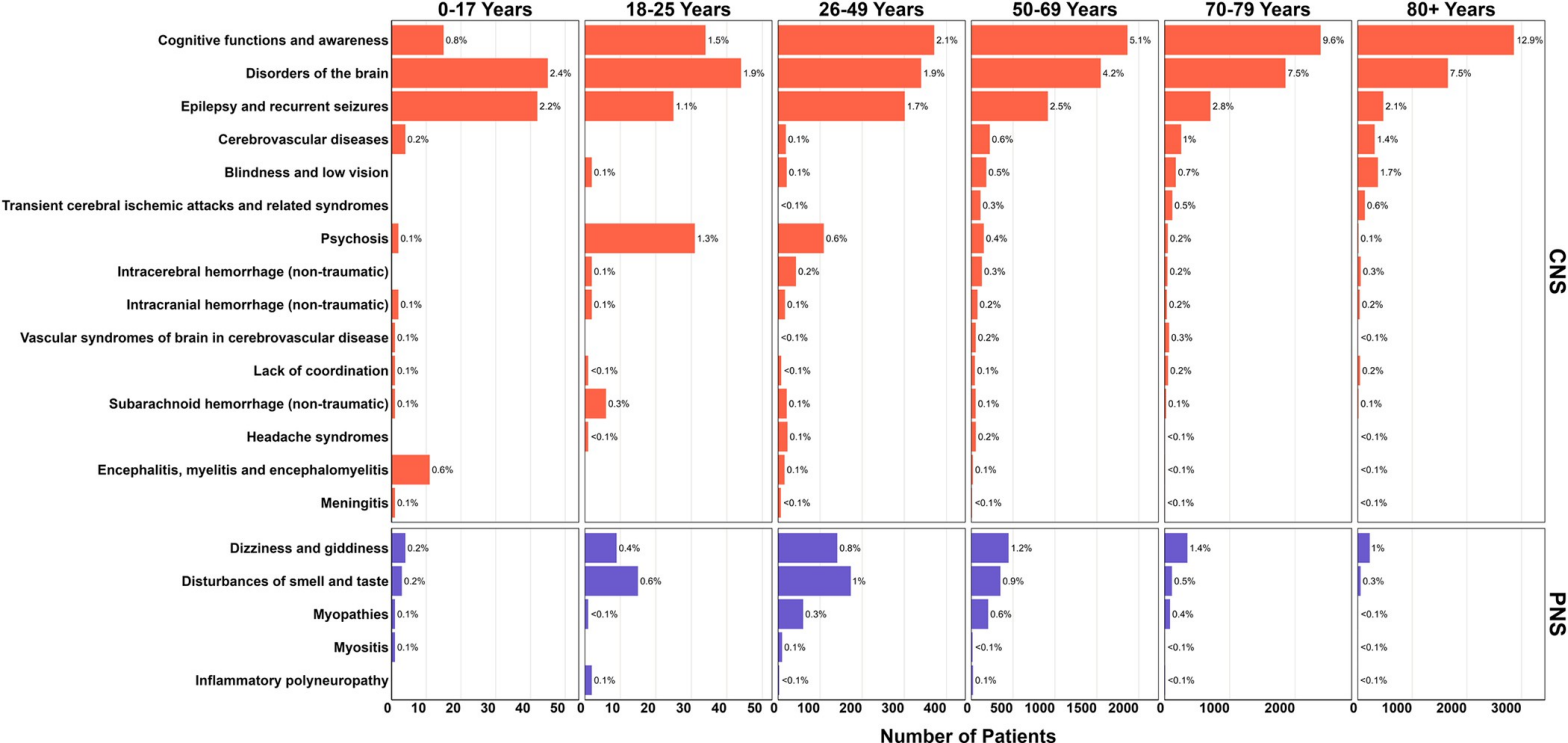
Frequency of neurological diagnosis codes by age group. For each age group, we report the total number and proportion of patients who had the associated ICD-10 code. Neurological diagnoses are listed in descending order of overall frequency. Please refer to S1 Fig for the incidence of severe COVID-19 status and mortality as stratified by concurrent neurological status in adults and children. S5 Table details the total counts and percentages of patients with ICD-10 (and ICD-9 codes) as stratified by adult and pediatric populations.

During the index COVID-19 hospitalization, 14,938 (14%) of adult patients had at least one CNS diagnosis code and 2,772 (3%) of adult patients had at least one PNS diagnosis code (S9 Table). The frequency of neurological diagnoses in the pediatric population was much lower: 163 (7%) pediatric patients had at least one CNS diagnosis, while only 16 (<1%) had a PNS diagnosis (S10 Table). The most frequent CNS diagnoses among *all* patients included symptoms and signs involving cognitive functions and awareness (*e*.*g*., altered mental status) (7.1%), disorders of the brain (*e*.*g*., encephalopathy, post viral fatigue syndromes, and anoxic brain injury) (5.2%), and epilepsy and recurrent seizures (2.3%). The most frequent PNS diagnoses among *all* patients included “dizziness and giddiness” (1.2%), “disturbances of smell and taste” (0.8%) and “myopathies” (0.4%). The most frequent diagnoses largely remained consistent when stratified by age groups or clinical outcome (Fig 3, S1 Fig, S5 Table). S2 and S5 Tables provide more detailed descriptions of the specific diagnosis codes for classifying the neurological diagnosis as either CNS or PNS.

#### Pre-existing comorbidity burden

**Fig 4:**
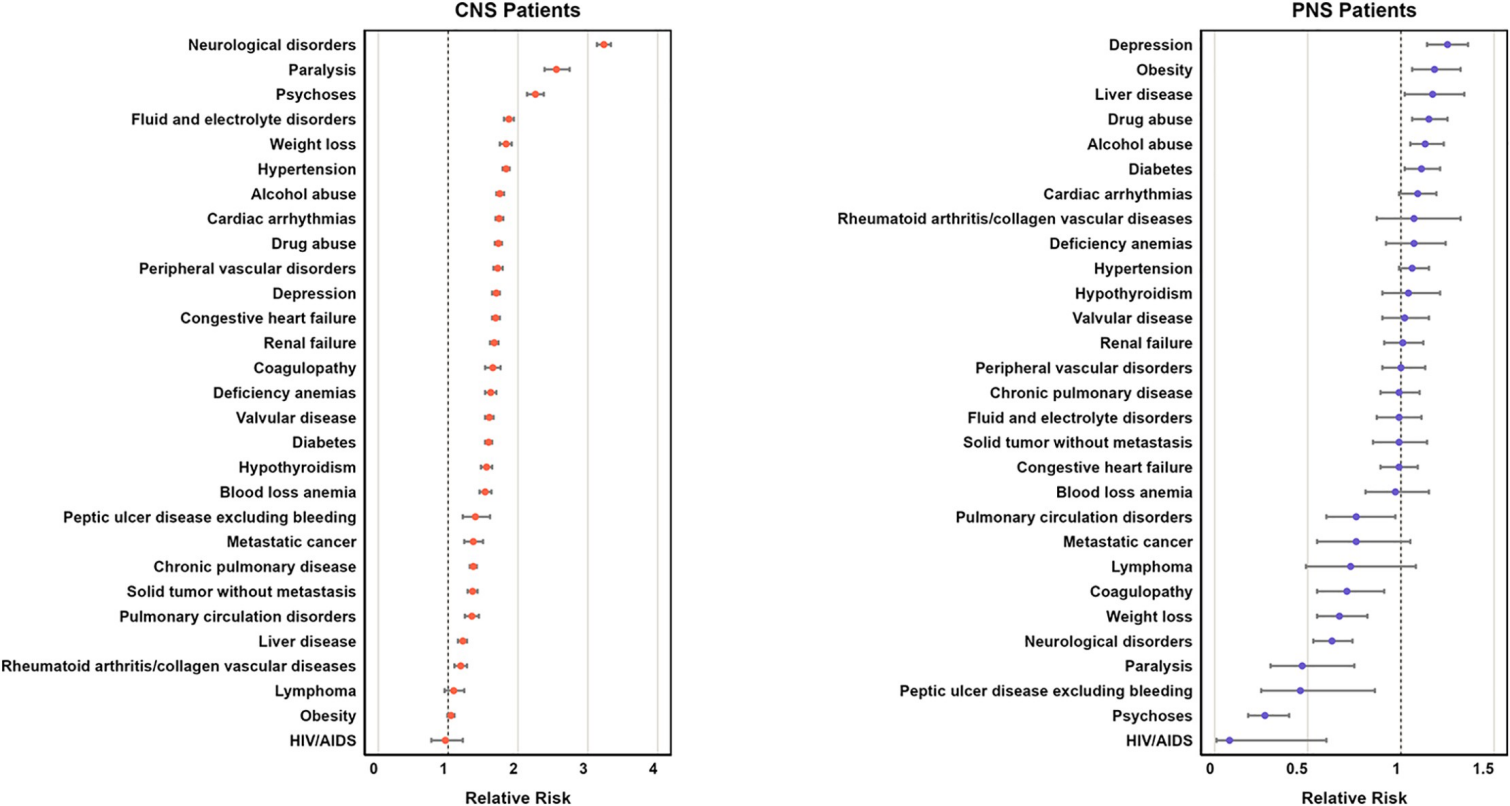
Relative risk of a neurological diagnosis in the adult patient population during acute COVID-19 hospitalization for each pre-admission health condition. We calculated the relative risks (with 95% confidence intervals) of any central nervous system (CNS) diagnosis (**A**) and any peripheral nervous system (PNS) diagnosis (**B**) during acute COVID-19 hospitalization for each pre-existing health condition (in the Elixhauser Comorbidity Index) by dividing the proportion of patients with the condition who developed a neurological diagnosis (CNS or PNS), by the number of patients without the condition who developed a neurological diagnosis. Pediatric patients were excluded from the analysis due to their low frequency of pre-admission health conditions. S3–S4 Tables provide detailed descriptions of the ICD codes comprising each component of the Elixhauser Comorbidity Index.

**Fig 5:**
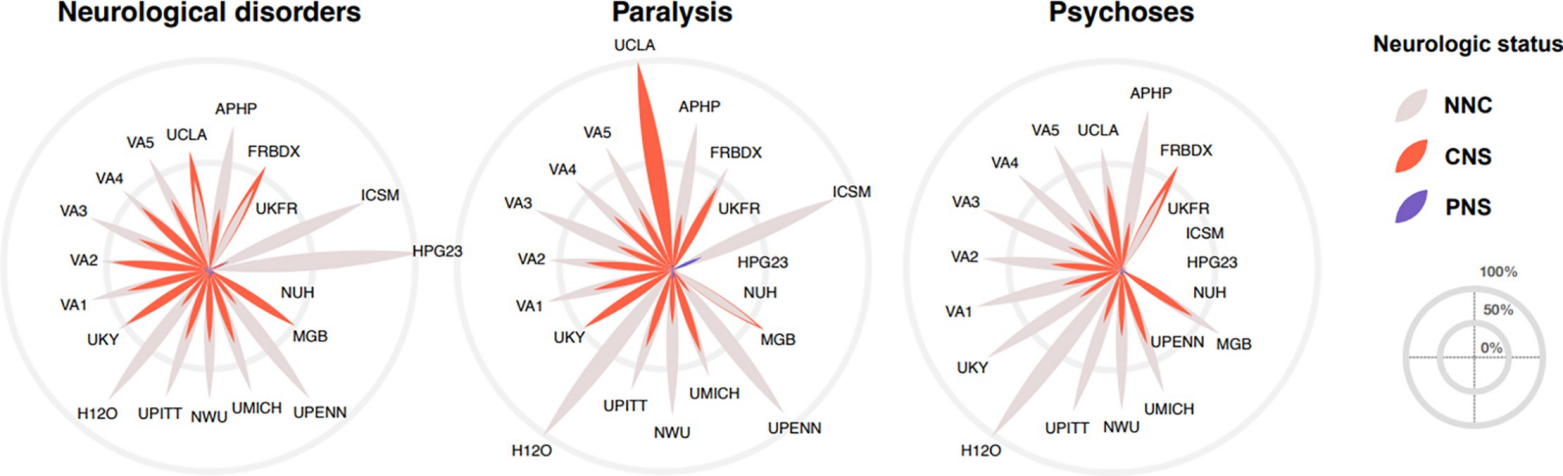
Pre-admission health conditions with the highest risk for a central nervous system diagnosis during acute COVID-19 hospitalization. Each petal plot represents the normalized distribution (%) of patients with a pre-admission health condition (as components of the Elixhauser Comorbidity Index: *e*.*g*., neurological disorders, paralysis, or psychoses) at each healthcare system for each neurological status during acute COVID-19 hospitalization: no neurological condition (NNC), central nervous system (CNS) diagnosis, and peripheral nervous system (PNS) diagnosis. Each nested petal represents a healthcare system. The colors within each petal are sorted based on their value: the outermost color indicating the neurological status with the highest portion of patients and the innermost color indicating the neurological status with the lowest portion at each healthcare system. With *n*_*k*_ indicating the number of patients from healthcare system *k* for condition *c*, and *n*_*1*_, *n*_*2*_, and *n*_*3*_ indicating the number of patients from the NNC, PNS, and CNS group, respectively, we summed patients at each system as *n*_*k*_ *= n*_*1*_*+n*_*2*_*+n*_3_. Missing petals indicate no patient for the pre-admission health condition at a healthcare system (*n*_*k*_ *= n*_*1*_ *= n*_*2*_ *= n*_3_ = *0*). A petal containing only one color indicates that patients with a given pre-admission health condition *c* at that healthcare system all had the same neurological status during acute COVID-19 hospitalization (*e*.*g*., *n*_*k*_ *= n*_*1*_ or *n*_*k*_ *= n*_*2*_ or *n*_*k*_ *= n*_*3*_). Using the pre-admission health condition ‘paralysis’ as an example, all patients with pre-admission paralysis at UCLA had a CNS diagnosis during acute COVID-19 hospitalization. Pediatric patients were excluded from the analysis due to low frequency of children with pre-admission health conditions.

In the adult population, the pre-existing comorbidity burden (pre-admission ECI score) was the highest in the CNS group (mean = 1.4, SD = 2), followed by the PNS group (mean = 0.6, SD = 1.7), and the lowest in the NNC group (mean = 0.2, SD = 0.5) (p = 0.074) (S9 Table). When assessing the relative risk (RR) of a CNS or PNS diagnosis during acute COVID-19 hospitalization, we found most pre-existing conditions increased the RR of a CNS diagnosis during COVID-19 hospitalization (Fig 4, S6 Table). In contrast, most pre-existing conditions decreased or had no effect on the RR of a PNS diagnosis during COVID-19 hospitalization. In adults, the pre-existing conditions with the highest risk for a CNS diagnosis during COVID-19 hospitalization included pre-existing neurological conditions (RR = 3.23, 95%CI: 3.13–3.33), paralysis (RR = 2.55, 95%CI: 2.38–2.74), and psychosis (RR = 2.25, 95%CI: 2.13–2.37) (Fig 4A) despite wide variation in the proportion of patients with pre-existing conditions across healthcare systems (Fig 5, S3 Fig). S4 Table lists the diagnoses captured by “neurological disorders” in ECI, which largely pertain to neurodegenerative diseases. The pre-existing conditions with the highest risk for a PNS diagnosis during acute COVID-19 hospitalization in adults included depression (RR = 1.25; 95%CI: 1.14–1.36), obesity (RR = 1.18, 95%CI: 1.06–1.32), and liver disease (RR = 1.17; 95%CI: 1.02–1.34) (Fig 4B, S6 Table).

In the pediatric population, the frequency of pre-existing health conditions was overall lower than that of adults. Notably, the mean pre-admission ECI score was higher in the CNS (mean = 4.4, SD = 9.2) and PNS (mean = 2.5, SD = 3.6) groups than the NNC group (mean = 0, SD = 0) (S10 Table). The overall counts and percentages of both adult and pediatric patients with pre-existing health conditions as stratified by neurological status are detailed in S3 Fig and S7 Table. For example, 25.2% of pediatric patients (as compared to 23.2% of adult patients) in the CNS group had a pre-existing neurological disorder, and likewise 11% of the pediatric patients (as compared to 3.4% of adult patients) in the CNS group had pre-existing paralysis (S7 Table).

In the adult population, concurrent neurological diagnoses assessed during acute COVID-19 hospitalization often appeared to be new onset since the mean numbers of pre-admission CNS and PNS diagnosis codes were 0 (S9 Table). Similarly, in children, the mean number of pre-admission PNS diagnosis codes was also 0. However, children with a CNS diagnosis had a slightly higher mean number of pre-admission CNS diagnoses (mean = 1.9, SD = 3.9) compared to children in the PNS (mean = 0, SD = 0) or NNC groups (mean = 0.1, SD = 0.2), though these differences were not significant (S10 Table).

### Primary clinical outcomes in adults with a neurological diagnosis during acute COVID-19 hospitalization: Meta-analysis results

**Fig 6:**
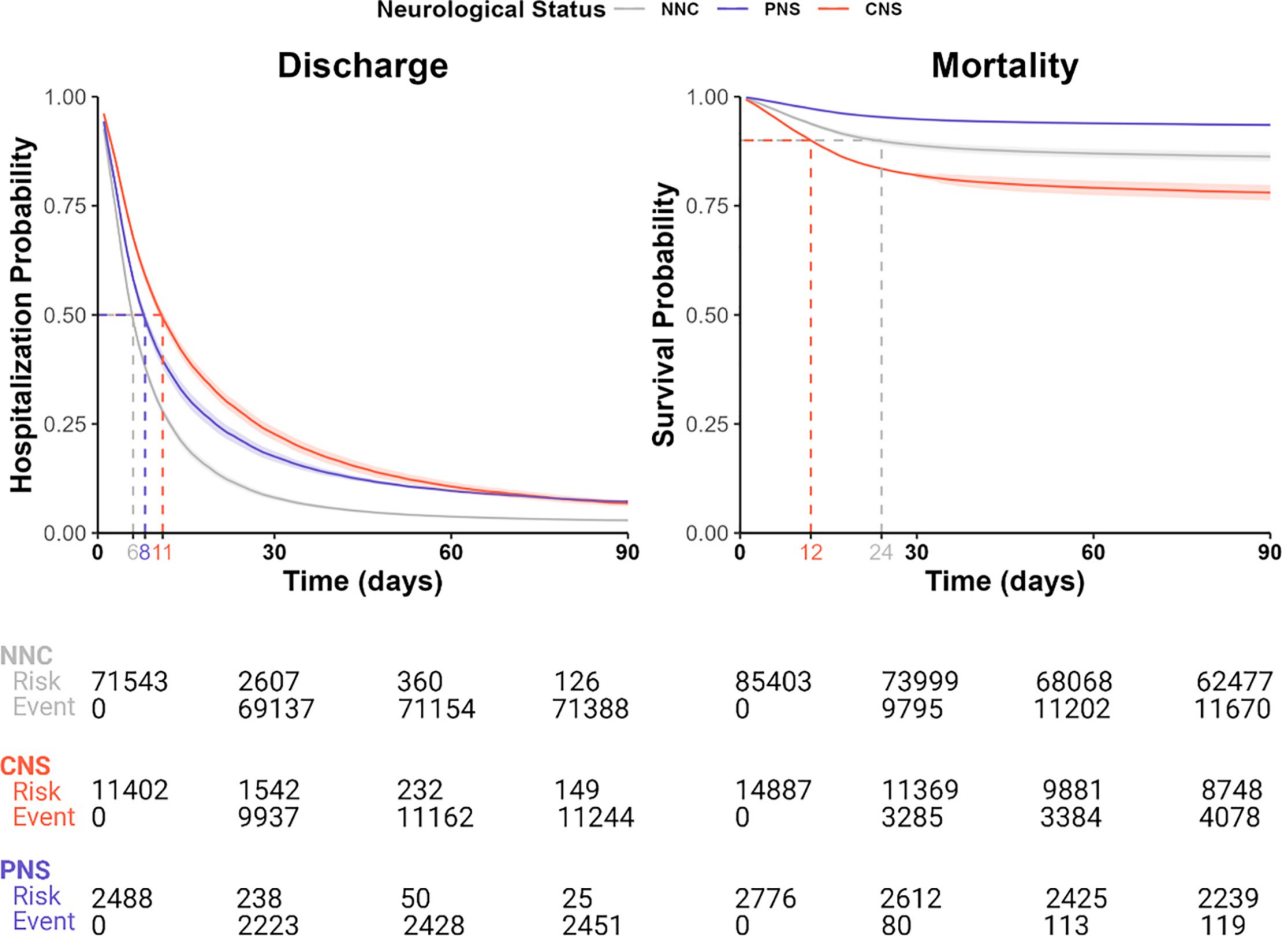
Covariate-adjusted Kaplan-Meier survival analysis to evaluate the time to event of each clinical endpoint stratified by neurological status during acute COVID-19 hospitalizations in adults. At each healthcare system, we estimated covariate-adjusted Kaplan-Meier time to event curves for each health outcome and neurological group. Specifically, for each outcome, we fit the Cox proportional hazards model to the patient cohort and estimated each patient’s survival rate, which is 1 minus event rate. Importantly, the survival rate was estimated for each patient by holding each patient’s covariates constant except for the neurological status. Thus, we estimated the survival rate for each neurological group, independent of the effect of additional covariates. Lastly, for each neurological group, we averaged all patients’ estimated survival rates to generate the overall survival curve. Survival curves from each healthcare system were combined using a random-effects meta-analysis weighted by the inverse of the variance derived at each participating healthcare system. Two healthcare systems (NUH and UKFR) were excluded from the meta-analysis due to their low frequency of neurological diagnoses (< 1% of adult patients). For the discharge outcome, we demarcated the median hospital stay in days for each neurological group. Due to a lower event rate for mortality, we demarcated the survival probability of the 90^th^ percentile for both the CNS and NNC groups. As the PNS group had <10% mortality, its survival probability was not demarcated. The table depicts the estimated total number of patients across all healthcare systems who were at risk at 0, 30, 60 and 90-day timepoints, where day 0 is the index date of the first COVID-19 hospitalization. Risk = the total number of patients who were still at risk for the event at a given time point. Event = total cumulative number of patients who experienced discharge or mortality by a given time point.

Using adults in the NNC group as the reference, adult patients with at least one CNS diagnosis during acute COVID-19 hospitalization had a lower risk of hospital discharge (HR = 0.54, 95%CI: 0.48–0.60, p < .001) (Table 2, Fig 6, S2 Fig). A lower risk of hospital discharge should be interpreted as longer hospital stay, as patients in the CNS group had a longer median hospital stay before discharge as compared to patients in the NNC group (11 days vs 6 days) (Fig 6). Adults with at least one PNS diagnosis during COVID-19 hospitalization also had a lower risk of hospital discharge (HR = 0.70, 95%CI: 0.60–0.82; p < .001) and longer median hospital stay before discharge (8 days vs 6 days) as compared to the NNC group.

**Table 2:** Random-effects meta-analysis of the risk of adverse clinical outcomes in adults with concurrent CNS or PNS diagnosis during the acute COVID-19 hospitalization from the Cox-proportional hazard models locally run at each healthcare system.

Clinical Outcome ^1^	Neurological Status ^2^	Hazard Ratio 95% CI ^3^	P-value ^4^
Discharge	CNS	0.54 (0.48, 0.60)	< .001*
	PNS	0.70 (0.60, 0.82)	< .001*
Mortality	CNS	1.78 (1.50, 2.11)	< .001*
	PNS	0.46 (0.38, 0.56)	< .001*

Notes

1. Please refer to Fig 6 for sample size of the analysis of each outcome. We excluded two healthcare systems (NUH and UKFR) from the meta-analysis due to low frequency of neurological diagnoses (< 1% of adult patients).

2. Concurrent neurological status during the acute COVID-19 hospitalization was categorized based on having at least one diagnosis code for central nervous system (CNS) or peripheral nervous system (PNS) diagnosis versus no neurological condition (NNC).

3. The hazard ratio above 1 indicates an increased risk for an event using the group of patients without any neurological condition during the acute COVID-19 hospitalization as the reference.

4. P-value with asterisk (*) denotes significance below the Bonferroni-corrected threshold for multiple testing (p-value threshold of < .013) given four separate tests.

5. An equivalent meta-analysis with pediatric patients could not be performed due to the small sample size of neurological diagnoses.

The CNS group had a greater risk of death than the NNC group (HR = 1.78, 95%CI: 1.50–2.11, p < .001) (Table 2, Fig 6, S2 Fig). 10% of the adult patients in the CNS group died within 12 days, compared to 24 days in the NNC group (Fig 6). In contrast, the PNS group had a lower risk of death (HR = 0.46, 95%CI: 0.38–0.56, p < .001). Indeed, 6.9% of PNS patients (versus 31.4% CNS patients) in the study cohort died (S9 Table).

### Supplementary analyses

The estimated concordances of the locally run Cox proportional hazard models were similar among models with increasing censor cutoff windows (30, 60 and 90 days after the index COVID-19 hospital admission) and across different adjustment methods for pre-existing comorbidities (S4 Fig). The hazard ratios derived from random-effects meta-analysis were also similar across censor windows and pre-existing comorbidity adjustment methods (S5 Fig).

Among the participating healthcare systems, we observed greater frequency of co-occurrence between a CNS diagnosis and severe COVID-19 than that of co-occurrence between a PNS diagnosis and severe COVID-19 based on the PMI analysis (S8 Table).

When comparing the estimated hazard ratios derived from a random-effects meta-analysis assessing age groups and the clinical outcomes, we observed an incremental increase in the risk of longer hospital stay and death with older age groups (S6 Fig). Indeed, in our cohort, patients 80 years and older were 13 times more likely to die than patients in the reference age group of 18–25 years (HR = 13.4, 95% CI: 9.6–18.7). As another example, patients in the age group of 50–69 years and older groups were twice more likely to have longer hospital stay (*i*.*e*., half as likely to be discharged) when compared to patients in the 18–25 years age group.

## Discussion

Leveraging a large, geographically diverse, multinational cohort, we investigated the clinical outcomes of hospitalized COVID-19 patients with concurrent neurological diagnoses. Our unique study design adopted a federated framework in which local clinician and informatics experts at each participating healthcare system ensured critical EHR data quality control while preserving data confidentiality. The consistent findings across geographically diverse healthcare systems support the generalizability and validity of our study.

To examine the association between neurological status during acute COVID-19 hospitalization and the risk of prolonged hospital length of stay and mortality in adults, we compared patients having CNS or PNS diagnoses during COVID-19 hospitalization with those without (NNC) both at the individual healthcare system level and collectively using meta-analysis. Our main finding was that adults with a CNS diagnosis had a greater risk for longer hospital stay and mortality during acute COVID-19 hospitalization than the NNC group after accounting for confounders, consistent with prior reports [6,9,11,42,43]. Our study differentiated from prior studies not only by examining a large multinational cohort of geographically diverse patient populations but also separately comparing the outcomes of two distinct groups of neurological conditions, CNS versus PNS, both using NNC as the control. This categorization allowed a broad comparison of the clinical outcomes of the distinct central versus peripheral nervous system pathology during acute COVID-19 while greatly reducing multiple hypothesis testing burden.

Indeed, the distinction between concurrent CNS and PNS involvement during acute COVID-19 hospitalization was clinically meaningful since the two groups of adults differed in clinical outcomes. One plausible explanation for the longer hospital stay of the CNS group was that these patients were more likely to experience severe disease, relative to the PNS (or NNC) group. Prior studies reported prolonged hospital course as secondary to complications of COVID-19 rather than active or persistent SARS-CoV-2 infection [44–47]. We also found that CNS diagnoses co-occur more frequently with other clinical characteristics of severe COVID-19 such as a diagnosis of pneumonia and/or acute respiratory distress syndrome, need for mechanical ventilation, sedation, and/or medication administration for shock [37] than PNS diagnoses (S8 Table).

Adults in the CNS group had a higher risk of death, compared to patients in the NNC group. In comparison, patients in the PNS group had a lower risk of death than the NNC group. The difference in the risk of mortality between the CNS and PNS groups may have at least two explanations. First, the most frequently observed CNS diagnoses in this study, including disorders of consciousness (*e*.*g*., altered mental status), disorders of the brain (*e*.*g*., encephalopathy), and seizure have all previously been reported in association with a greater risk of death [9,42,48,49]. Thus, the CNS group in this study likely included more diagnoses associated with critical illness. For instance, acute encephalopathy is common among hospitalized COVID-19 patients and known to cause greater need for critical care, intubation, severe disability and 30-day mortality [9,14,50]. Acute encephalopathy falls under the parent ICD-10 code G93, which was the second most frequently observed neurological ICD-19 code in our cohort. Acute encephalopathy could result from systemic dysfunction or neuropathologies such as hypoxic ischemic brain injury [44]. In contrast, the PNS diagnoses in our cohort, largely comprised less severe diagnoses (*e*.*g*., anosmia and dysgeusia, myopathies), likely contributing to the observed inverse association between a PNS diagnosis and mortality. Second, ascertainment bias might contribute to the lack of significant association between concurrent PNS diagnosis and the potential for severe disease leading to mortality [43,51,52]. For example, EHR data might contain incomplete documentation of certain PNS diagnoses such as anosmia or dizziness in critically ill patients who would be less capable of reporting such symptoms and whose diagnostic signs might not be easily recognizable and used for diagnosis coding. In support of our findings, prior studies also reported lower risk of mortality in hospitalized COVID-19 patients with anosmia, ageusia, and syncope, though rationale beyond ascertainment bias is unknown [43,51,52].

In our adult patient population, we also assessed the relative risk of developing a neurological diagnosis during acute COVID-19 hospitalization for each of the pre-existing health conditions that constitute the ECI. While pre-existing neurological conditions had been reported to increase the risk of new neurological complications during acute COVID-19 [53], our study uniquely identified the pre-existing health conditions that increased (or decreased) the risk of concurrent CNS and PNS diagnoses in adults during acute COVID-19 hospitalization. Patients in the CNS group had more pre-existing health conditions than the PNS group. Depression, drug and alcohol abuse, and diabetes were associated with higher risk of both CNS and PNS diagnoses during COVID-19 hospitalization. Interestingly, certain pre-existing conditions were associated with CNS and PNS diagnoses in opposite directions. For instance, coagulopathy, peptic ulcer disease, pulmonary circulation disorders and weight loss were associated with higher risk of CNS diagnosis, but with lower risk of PNS diagnosis. Likewise, pre-existing neurological conditions (*i*.*e*., “neurological disorder” that includes primarily neurodegenerative diseases, “paralysis”, and “psychoses”), which all exhibited the strongest association with CNS diagnosis, were associated with a lower risk of PNS diagnosis during acute COVID-19 hospitalization. These findings again highlight the importance of separately assessing CNS and PNS diagnoses in COVID-19.

Finally, a key strength of the study is the inclusion of children hospitalized for acute COVID-19, which we analyzed separately from adults. It is reassuring to replicate the lower prevalence of acute COVID-19 hospitalization in children than adults [54]. However, pediatric neurological manifestations, particularly CNS involvement (7.4% in children as compared to 14.4% in adults), were not infrequent in our study. Indeed, across the participating healthcare systems that provided pediatric data, the frequency of children with any concurrent neurological diagnosis during acute COVID-19 hospitalization ranged from 0% to 28.6%. Similar to previous studies [15,16,18,55], the most frequent concurrent neurological diagnoses during acute COVID-19 hospitalization in our pediatric population included epilepsy and seizures, symptoms and signs involving cognitive function and awareness (*e*.*g*., altered mental status), and disorders of the brain (*e*.*g*., encephalopathy), while ischemic and hemorrhagic strokes were uncommon. Further, our findings were consistent with previous report of common pre-existing neurological conditions among children with neurological manifestations during acute COVID-19 [19]. The mean time to discharge were slightly longer for children in the CNS and PNS group than the NNC group, but these differences did not reach statistical significance as in adults. Importantly, while the overall mortality rate was low in our pediatric population (1.3% as compared to 18% in adults), a higher proportion of pediatric patients in the CNS group died as compared to the NNC group (5.8% vs. 1% in children, compared to 31.4% vs. 16.1% in adults). The CNS diagnoses most frequently associated with death in pediatric patients included diagnosis under disorders of the brain (*e*.*g*., encephalopathy, anoxic brain injury) (S1 Fig), consistent with prior reports [15,16]. Overall, our study replicates prior findings and adds to a limited body of literature evaluating the frequency of neurological manifestations and their health outcomes in hospitalized children during acute COVID-19.

### Limitations

The study has limitations primarily stemming from adopting a standardized strategy to analyze EHR data from geographically diverse multinational healthcare systems while preserving patient confidentiality. First, the use of parent ICD codes at the categorical level (*e*.*g*., ICD-10 G93: “disorders of the brain”) was a tradeoff to mitigate noise from variations in billing documentation and clinical practices and enable data harmonization across all participating multinational healthcare systems, which served as a key strength of the study.

Second, we had limited ability to confirm whether neurological diagnoses during acute COVID-19 hospitalization were new onset versus pre-existing. To reduce this concern, we adjusted for the occurrence of pre-existing CNS and PNS diagnoses in addition to the overall comorbidity burden in the survival analysis. It is worth noting that the mean numbers of pre-admission CNS and PNS diagnosis codes in our entire cohort was 0. As the 4CE consortium collected pre-existing conditions up to one year before the first COVID-19 hospitalization (as a pragmatic decision to balance maximizing data collection versus minimizing past medical conditions that might not be relevant), undercounting of pre-existing diagnoses might still occur, particularly in situations where patients received care at outside facilities prior to COVID-19 hospitalization.

Third, vaccination data capture for the study population was likely incomplete as the existing EHR data may not incorporate vaccinations administered at external hospitals, clinics, or pharmacies [56]. Moreover, the availability and distribution of vaccines across our participating multinational healthcare systems were not standardized [57]. While vaccination status influences the risk of hospitalization in acute COVID-19, our study design was standardized to include *only* hospitalized patients. By evaluating only hospitalized patients in the pre-omicron period, we reduced the potential unmeasured confounding from vaccination status. In future studies, we will examine the outcomes of neurological manifestations during acute COVID-19 in sub-populations with confirmed vaccination status and across time periods to adjust for the increasing frequency of vaccination over time.

Fourth, the sample size of children hospitalized with acute COVID-19 was relatively modest (n = 2,198) but comparable to the only other known multinational study of neurological manifestations in both adults and children during acute COVID-19 hospitalization [20]. Reassuringly, the frequency of neurological diagnoses in children versus adults are mostly consistent between our two studies. Overall, the inclusion of pediatric patients enhanced the diversity of our study population, permitted validation of the pediatric frequency of neurological conditions, and provided a basis for future studies.

Fifth, the available data collection from the 21 participating healthcare systems does not support a more rigorous subgroup analysis by age groups. While nearly 80% of the study population was ≥ 50 years of age, we acknowledge the possibility that the limited variation in the age of our adult study cohort may have introduced biases that could possibly reduce the generalizability of our findings. Study cohorts with even greater variation in age than the current dataset would be necessary to examine the interaction between age and neurological diagnoses with respect to adverse outcomes in hospitalized acute COVID-19 patients.

Finally, the pre-defined key aim of our study was to distinguish the risks between central and peripheral nervous system diagnoses during acute COVID-19 hospitalization. Indeed, our main findings of the higher risk for prolonged hospital stay and mortality among hospitalized patients with acute COVID-19 and a concurrent central nervous system diagnosis are still clinically relevant. While the analysis of more granular neurological groups or individual neurological diagnoses could be informative, additional stratified analyses would reduce statistical power (given the fewer events per group) to draw clinically meaningful conclusions. To properly conduct stratified analyses leveraging clinically meaningful neurological diagnosis groups or individual neurological diagnosis from the EHR data, we would require substantially larger cohorts for future analyses.

## Conclusion

In this large multinational and geographically diverse cohort with a federated framework for leveraging locally curated EHR data for clinical discovery, we analyzed the clinical outcomes of both hospitalized adult and pediatric COVID-19 patients with concurrent CNS or PNS diagnosis. Adults with concurrent CNS diagnosis during COVID-19 hospitalization harbored greater burden of pre-existing health conditions and had greater risk of poor clinical outcomes (prolonged hospitalization and death) when compared to those with PNS diagnosis or no neurological diagnosis. We observed similar patterns in children though the overall low frequency of events prohibited a formal survival analysis. Our study underscores the need for careful evaluation and prompt treatment of neurological conditions, particularly those involving the CNS, in hospitalized COVID-19 patients. Future investigation of the impact of the pre-existing and concurrent neurological conditions during the acute phase of COVID-19 on post-acute sequelae of COVID-19 will be crucial for both adults and children.

## Supporting information

S1 Fig **Frequency of concurrent neurological diagnoses among hospitalized children (A) and adults (B) with acute COVID-19 who reached severe status or died.** For mortality and severity, we reported the total number and proportion of patients who met the clinical endpoint and had the associated neurological diagnosis code (*i*.*e*., ICD-10 code). Neurological diagnoses are listed in descending order of overall frequency.
(PDF)

S2 Fig Meta-analysis of the risk of adverse clinical outcomes stratified by concurrent neurological status and outcome during acute COVID-19 hospitalizations in adults. Adverse outcomes include lower risk of hospital discharge and higher risk of mortality. Neurological status during COVID-19 hospitalization included any central nervous system (CNS) diagnosis (**A, C**) or any peripheral nervous system (PNS) diagnosis (**B, D**). Black circles indicate the local healthcare system-level hazard ratio derived from the Cox proportional hazards model. The red diamond represents the pooled effect size derived from the random-effects meta-analysis. The effect size and associated p-value derived from meta-analysis are reported in Table 2 of the main text. We also report the following metrics: I2 (95% CI), the estimated proportion of variance due to differences among healthcare systems; (Tau) τ^2^, the between-healthcare system variance; Prediction Interval, the predicted effect size if we were to add a new healthcare system to the analysis. We excluded two adult healthcare systems (NUH and UKFR) from the meta-analysis due to low frequency of neurological diagnoses in their patient populations (< 1% of adult patients).
(PDF)

S3 Fig Adult and pediatric pre-admission health conditions across healthcare systems. Each grouped stacked bar chart represents the normalized distribution (%) of adults (**A**) or children (**B**) with a specific pre-admission health condition (that collectively constitute the Elixhauser Comorbidity Index) at each healthcare system for each neurological status during acute COVID-19 hospitalization. Neurological status included no neurological condition (NNC), central nervous system (CNS) diagnosis, and peripheral nervous system (PNS) diagnosis. Each stacked bar represents a healthcare system. With nk indicating the number of patients from healthcare system k for pre-admission health condition c, and n1, n2, and n3 indicating the number of patients from the NNC, PNS, and CNS group, respectively, we summed patients at each system as nk = n1+n2+n3. Missing bars indicate no patients for the given pre-admission health condition at a healthcare system (nk = n1 = n2 = n3 = 0). A bar with a single color indicates that patients with a given pre-admission health condition c at that healthcare system all had the same neurological status (e.g. nk = n1 or nk = n2 or nk = n3).
(PDF)

S4 Fig Concordance of Cox proportional hazard models. Cox proportional hazard models were constructed using three censor cutoff periods (30, 60, and 90 days) and three methods of adjusting for pre-admission health conditions (*i*.*e*., pre-existing comorbidity burden): (1) the inclusion of the 29 individual covariates (*i*.*e*., health conditions) comprising the Elixhauser Comorbidity Index, (2) the Elixhauser summary score, and (3) the top 10 principal components computed from logistic principal component analysis (LPCA). Violin plots represent the distribution of concordance across healthcare systems (each black dot representing the concordance from a specific healthcare system).
(PDF)

S5 Fig Hazard ratios of a random effects meta-analysis of locally estimated Cox proportional hazard models. Cox proportional hazard models were constructed using three censor cutoff periods (30, 60, and 90 days) and three methods of adjusting for pre-admission health conditions (or comorbidity burden): (1) the inclusion of the 29 individual covariates (health conditions) comprising the Elixhauser Comorbidity Index, (2) the Elixhauser summary score, and (3) the top 10 principal components computed from logistic principal component analysis (LPCA). A random effects meta-analysis was performed to determine the pooled hazard ratio (HR) and 95% confidence intervals of each outcome. The meta-analysis was performed separately for central nervous system (CNS) and peripheral nervous system (PNS) diagnosis during acute COVID-19 hospitalization to evaluate the risk of each outcome with respect to patients with no neurological condition (NNC).
(PDF)

S6 Fig Estimated hazard ratios by age group. For each outcome (hospital discharge, death), we conducted a random-effects meta-analysis to estimate the pooled hazard ratio and 95% confidence intervals for each age group using patients in the age group of 18–25 years as the reference group. The pooled hazard ratio for hospitalized patients with acute COVID-19 and concurrent central (CNS) and peripheral (PNS) nervous system diagnoses are also plotted as a reference. Dashed lines demarcate the hazard ratio at 1.
(PDF)

S1 Table Description of participating healthcare systems. (PDF)

S2 Table Descriptions of the parent ICD-10 diagnosis codes for classifying concurrent central or peripheral nervous system manifestations during acute COVID-19 and their subcategory diagnosis codes. (PDF)

S3 Table List of the parent category ICD-10 diagnosis codes mapped to each condition of the Elixhauser Comorbidity Index and its associated weight. Notes: 1. Complicated and uncomplicated diabetes were combined as one condition. Likewise, complicated and uncomplicated hypertension were combined as one condition.
(PDF)

S4 Table Descriptions of the parent category ICD-10 diagnosis codes mapped to each condition of the Elixhauser Comorbidity Index. Notes: 1. Complicated and uncomplicated diabetes were combined as one condition. Likewise, complicated and uncomplicated hypertension were combined as one condition. 2. ICD-10 descriptions were curated using the *icd* R package [1]. Supplemental Citation 1. Wasey JO, Frank SM, Rehman MA. icd: Efficient Computation of Comorbidities from ICD Codes Using Sparse Matrix Multiplication in R. Journal of Statistical Software. 2018. Available: https://jackwasey.github.io/icd/articles/efficiency-prebuilt.pdf
(PDF)

S5 Table Major categories of ICD-10 and ICD-9 codes representing central or peripheral nervous system diagnoses in descending order of frequency observed in the adult population. Notes: 1. ICD-10 code R42 was listed as peripheral though certain dizziness symptoms could be of central origin. 2. ICD-10 code H54 and ICD-9 code 369 were listed as central but there could be peripheral causes for blindness.
(PDF)

S6 Table Relative risk (RR) of a concurrent central nervous system (CNS) or peripheral nervous system (PNS) diagnosis during acute COVID-19 hospitalizations with respect to each pre-admission health condition in descending order of the RR for the CNS diagnosis in adults.^1^ Notes: 1. We could not conduct similar analyses in children due to the low frequency of pre-admission health conditions in pediatric patients. Refer to S7 Table for the overall count and percentage of adult and pediatric patients with pre-admission health conditions as stratified by neurological status. 2. Refer to S3–S4 Tables for detailed descriptions of ICD codes comprising each component of the Elixhauser Comorbidity Index. 3. Complicated and uncomplicated diabetes were combined as one condition. Likewise, complicated and uncomplicated hypertension were combined as one condition.
(PDF)

S7 Table Count and percentage of adult and pediatric patients with pre-admission health conditions as stratified by concurrent neurological status during acute COVID-19 hospitalization ^1^. Notes: 1. Neurological status during acute COVID-19 hospitalization: central nervous system diagnosis (CNS), peripheral nervous system diagnosis (PNS), no neurological condition (NNC). 2. Refer to S3–S4 Tables for detailed descriptions of ICD codes comprising each component of the Elixhauser Comorbidity Index. 3. N = the total number of adult or pediatric patients with the pre-admission health condition; the corresponding percentage is out of the total adult or pediatric population. 4. Percentages in the NNC, CNS, and PNS columns reflect the percent of patients with the respective neurological status who have the indicated pre-admission health condition. 5. Complicated and uncomplicated diabetes were combined as one condition. Likewise, complicated and uncomplicated hypertension were combined as one condition.
(PDF)

S8 Table Pointwise mutual information (PMI) of a central nervous system (CNS) or peripheral nervous system (PNS) diagnosis co-occurring with severe COVID-19 disease during acute COVID-19 hospitalization. Notes: 1. We report each healthcare system’s total number of severe and neurological patients used to calculate the PMI at each healthcare system. PMI >0 indicates more frequent co-occurrence (between a CNS or a PNS diagnosis and severe COVID-19 status) than independent assumptions. 2. Severe COVID-19 status was based on previously published computable phenotypes, including diagnosis of pneumonia and/or acute respiratory distress syndrome, need for mechanical ventilation, sedation, and/or medication administration for shock [1]. 3. 95% confidence intervals were estimated using 500 bootstrapped samples. 4. Bold findings indicate statistically significant results. Supplemental Citation 1. Klann, J. G. et al. Validation of an internationally derived patient severity phenotype to support COVID-19 analytics from electronic health record data. *Journal of the American Medical Informatics Association* **28**: 1411–1420 (2021).
(PDF)

S9 Table Adult Population Characteristics. Notes: 1. Neurological status during acute COVID-19 hospitalization: NNC = No Neurological Condition; CNS = Central Nervous System diagnosis; PNS = Peripheral Nervous System diagnosis. 2. P-values were adjusted with the Benjamini-Hochberg method to control the false discovery rate when evaluating the distribution (categorical variables) or means (continuous variables) of characteristics stratified by neurological status. P-values < .05 were deemed significant and designated with asterisk (*). 3. Continuous variables reflect the overall cohort mean and standard deviation (SD) of the median values reported by each healthcare system. 4. We report the top 4 most frequent pre-admission health conditions (*i*.*e*., comorbidities) in the adult population. Please see S7 Table for all pre-admission health conditions stratified by neurological status during acute COVID-19 hospitalization. 5. We could not compute chi-square statistic for the mean number of pre-admission CNS codes, due to every healthcare system having a median value of 0.
(PDF)

S10 Table Pediatric Population Characteristics. Notes: 1. Neurological status during acute COVID-19 hospitalization: NNC = No Neurological Condition; CNS = Central Nervous System diagnosis; PNS = Peripheral Nervous System diagnosis. 2. P-values were adjusted with the Benjamini-Hochberg method to control the false discovery rate when evaluating the distribution (categorical variables) or means (continuous variables) of characteristics stratified by neurological status. P-values < .05 were deemed significant and designated with asterisk (*). 3. Continuous variables reflect the overall cohort mean and standard deviation (SD) of the median values reported by each healthcare system. 4. We report the top 4 most frequent pre-admission health conditions (*i*.*e*., comorbidities) in the pediatric population. Please see S7 Table for all pre-admission health conditions stratified by neurological status during acute COVID-19 hospitalization.
(PDF)

S1 Methods Protecting Patient Confidentiality. (PDF)

S2 Methods Logistic Principal Component Analysis. (PDF)
