# Correction: Neurological diagnoses in hospitalized COVID-19 patients associated with adverse outcomes: A multinational cohort study

**DOI:** 10.1371/journal.pdig.0000957

**Published:** 2025-07-22

**Authors:** Meghan R. Hutch, Jiyeon Son, Trang T. Le, Chuan Hong, Xuan Wang, Zahra Shakeri Hossein Abad, Michele Morris, Alba Gutiérrez-Sacristán, Jeffrey G. Klann, Anastasia Spiridou, Ashley Batugo, Riccardo Bellazzi, Vincent Benoit, Clara-Lea Bonzel, William A. Bryant, Lorenzo Chiudinelli, Kelly Cho, Priyam Das, Tomás González González, David A. Hanauer, Darren W. Henderson, Yuk-Lam Ho, Ne Hooi Will Loh, Adeline Makoudjou, Simran Makwana, Alberto Malovini, Bertrand Moal, Danielle L. Mowery, Antoine Neuraz, Malarkodi Jebathilagam Samayamuthu, Fernando J. Sanz Vidorreta, Emily R. Schriver, Petra Schubert, Jeffery Talbert, Amelia L. M. Tan, Byorn W. L. Tan, Bryce W. Q. Tan, Valentina Tibollo, Patric Tippman, Guillaume Verdy, William Yuan, Paul Avillach, Nils Gehlenborg, Gilbert S. Omenn, The Consortium for Clinical Characterization of COVID-19 by EHR (4CE), Shyam Visweswaran, Tianxi Cai, Yuan Luo, Zongqi Xia

Fig 2 and S1 Methods were uploaded incorrectly. Please see the correct Fig 2 and S1 Methods below.

**Fig 2:**
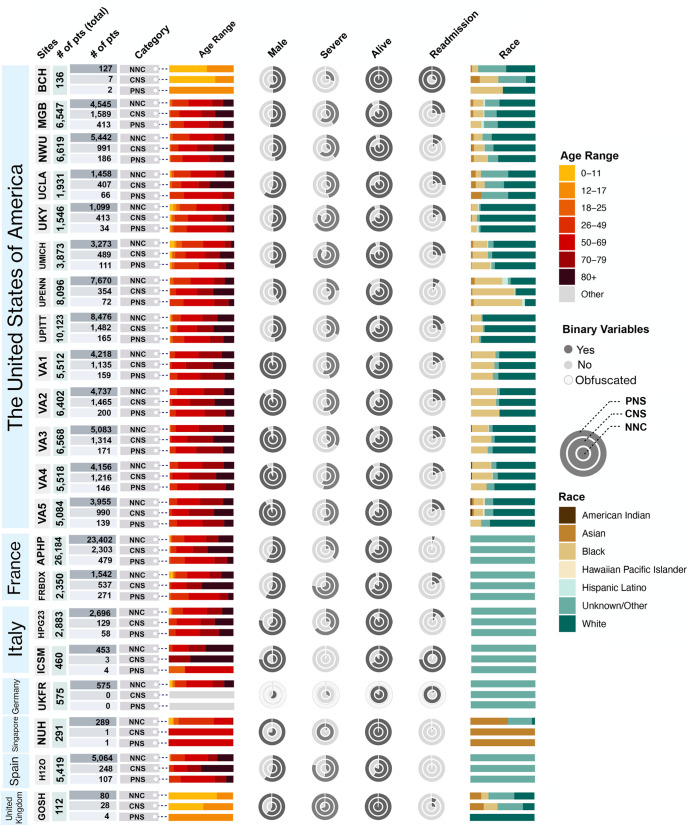
Demographic profile for each participating healthcare system arranged by country. Cohort-wise breakdown of the number of patients, age range, sex, severity status, mortality outcome, readmission status, and race at each healthcare system for each of the following neurological status during acute COVID-19 hospitalization: no neurological condition (NNC), central nervous system (CNS) diagnosis, and peripheral nervous system (PNS) diagnosis. Healthcare systems are arranged by country in descending order by the number of included participating healthcare systems. The stacked bar charts indicate the normalized distribution of age and race. The nested pie-charts are stratified by the neurological status with the darker portion representing the proportion of patients having the value of the binary variable for the given column header. See S1 Table for healthcare system abbreviations and S1 Methods for details on the obfuscation process that was applied to the depicted counts for the purposes of protecting patient confidentiality.

## Supporting information

S1 Methods Protecting patient confidentiality. (PDF)

## References

[1] Hutch MR, Son J, Le TT, Hong C, Wang X, Shakeri Hossein Abad Z, et al. Neurological diagnoses in hospitalized COVID-19 patients associated with adverse outcomes: A multinational cohort study. PLOS Digit Health. 2024;3(4):e0000484. doi: 10.1371/journal.pdig.0000484 38620037 PMC11018281

