# Supplementary material for: Neurological diagnoses in hospitalized COVID-19 patients associated with adverse outcomes: A multinational cohort study
Source: PLOS Digit Health. 2024 Apr 15;3(4):e0000484. doi: 10.1371/journal.pdig.0000484 (PMC11018281; doi:10.1371/journal.pdig.0000484)
Supplement: S1 Fig — Frequency of concurrent neurological diagnoses among hospitalized children (A) and adults (B) with acute COVID-19 who reached severe status or died. For mortality and severity, we reported the total number and proportion of patients who met the clinical endpoint and had the associated neurological diagnosis code (i.e., ICD-10 code). Neurological diagnoses are listed in descending order of overall frequency. (PDF) [file pdig.0000484.s001.pdf]

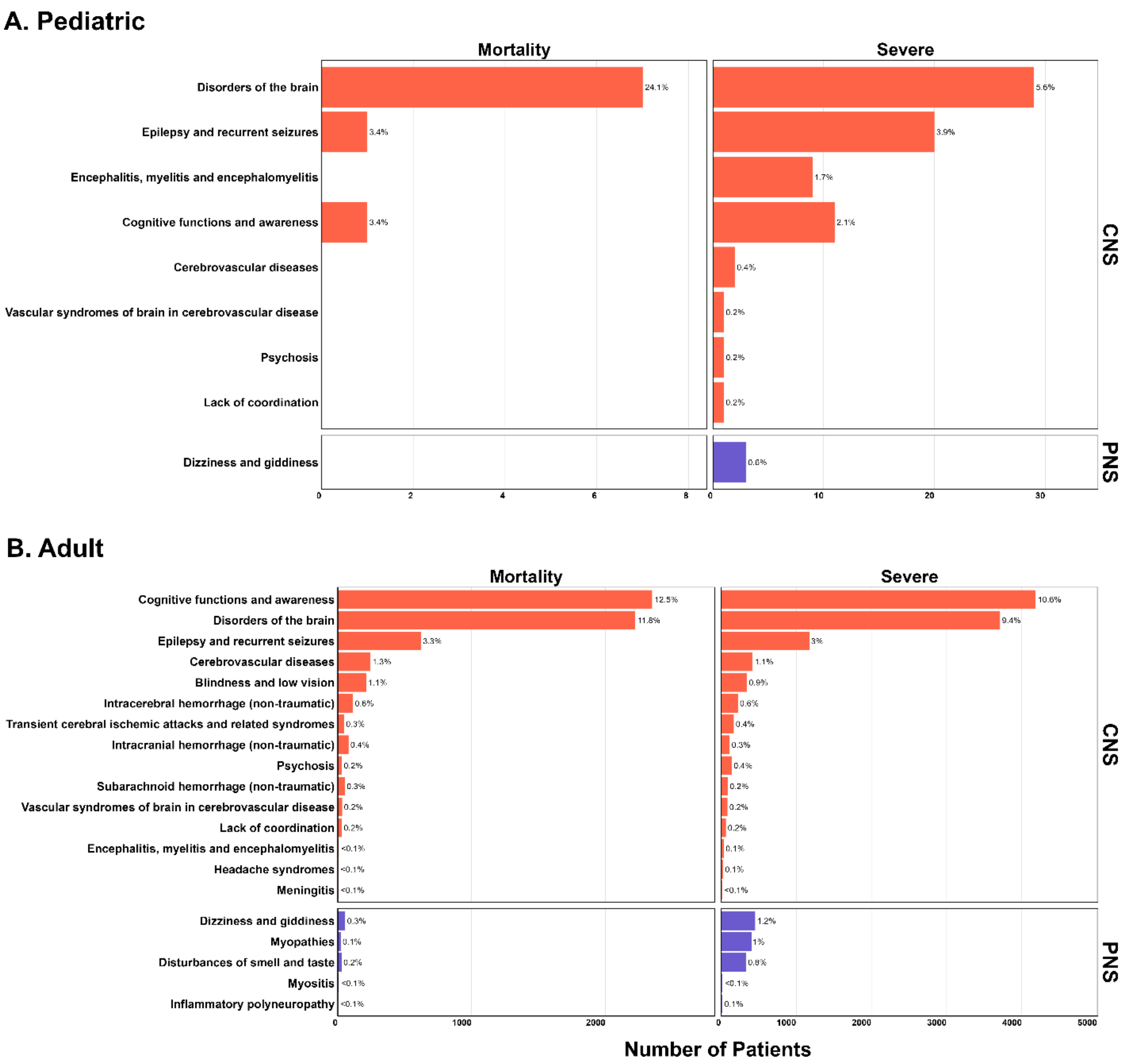


**S1 Fig. Frequency of concurrent neurological diagnoses among hospitalized children (A) and adults (B) with acute COVID-19 who reached severe status or died.** For mortality and severity, we reported the total number and proportion of patients who met the clinical endpoint and had the associated neurological diagnosis code (*i.e.,* ICD-10 code). Neurological diagnoses are listed in descending order of overall frequency.
