# Supplementary material for: Neurological diagnoses in hospitalized COVID-19 patients associated with adverse outcomes: A multinational cohort study
Source: PLOS Digit Health. 2024 Apr 15;3(4):e0000484. doi: 10.1371/journal.pdig.0000484 (PMC11018281; doi:10.1371/journal.pdig.0000484)
Supplement: S2 Fig — Adverse outcomes include lower risk of hospital discharge and higher risk of mortality. Neurological status during COVID-19 hospitalization included any central nervous system (CNS) diagnosis (A, C) or any peripheral nervous system (PNS) diagnosis (B, D). Black circles indicate the local healthcare system-level hazard ratio derived from the Cox proportional hazards model. The red diamond represents the pooled effect size derived from the random-effects meta-analysis. The effect size and associated p-value derived from meta-analysis are reported in Table 2 of the main text. We also report the following metrics: I2 (95% CI), the estimated proportion of variance due to differences among healthcare systems; (Tau) τ2, the between-healthcare system variance; Prediction Interval, the predicted effect size if we were to add a new healthcare system to the analysis. We excluded two adult healthcare systems (NUH and UKFR) from the meta-analysis due to low frequency of neurological diagnoses in their patient populations (< 1% of adult patients). (PDF) [file pdig.0000484.s002.pdf]

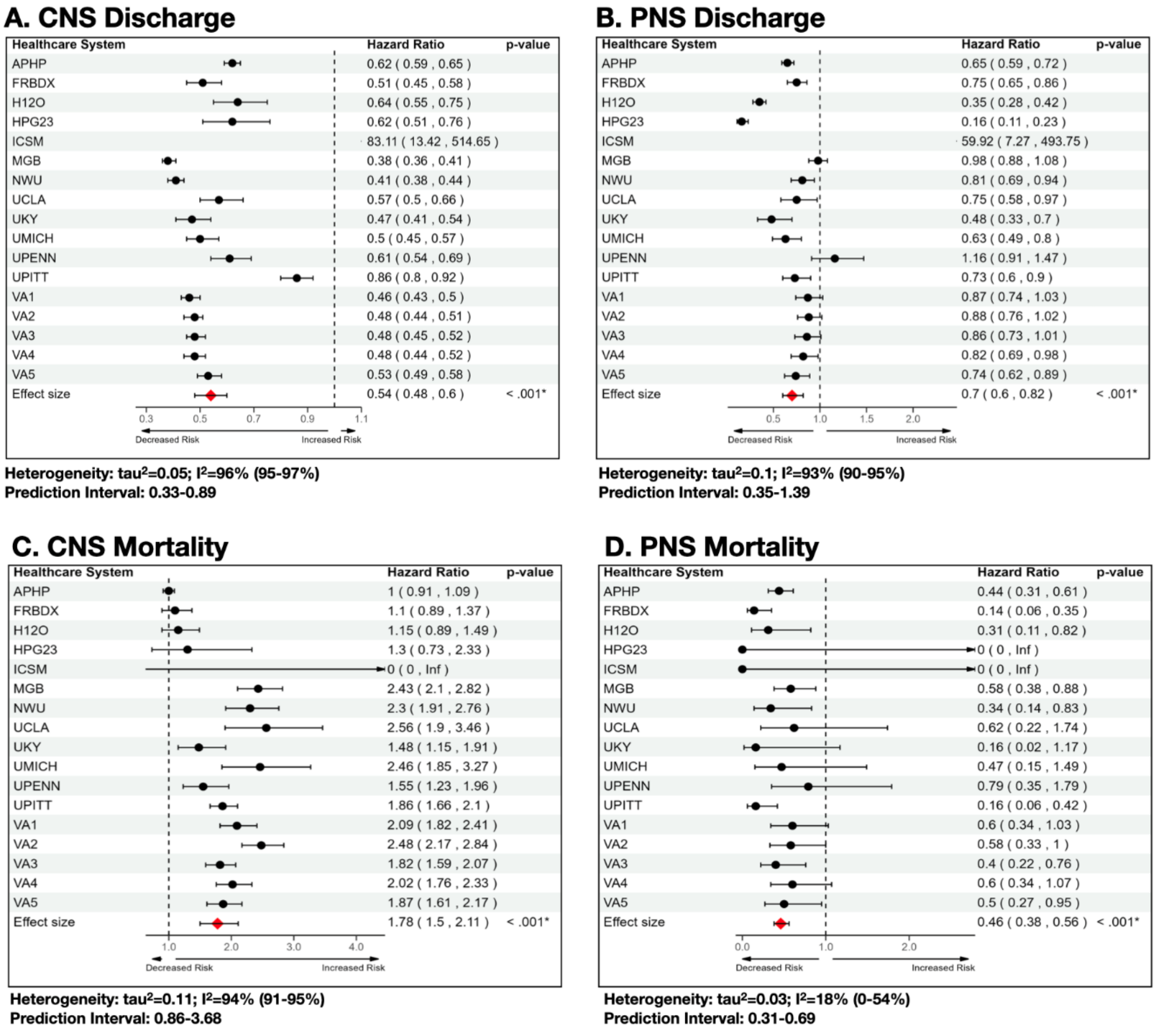

S2 Fig. Meta-analysis of the risk of adverse clinical outcomes stratified by concurrent neurological status and outcome during acute COVID-19 hospitalizations in adults. Adverse outcomes include lower risk of hospital discharge and higher risk of mortality. Neurological status during COVID-19 hospitalization included any central nervous system (CNS) diagnosis (**A, C**) or any peripheral nervous system (PNS) diagnosis (**B, D**). Black circles indicate the local healthcare system-level hazard ratio derived from the Cox proportional hazards model. The red diamond represents the pooled effect size derived from the random-effects meta-analysis. The effect size and associated p-value derived from meta-analysis are reported in Table 2 of the main text. We also report the following metrics: I2 (95% CI), the estimated proportion of variance due to differences among healthcare systems; (Tau) τ^2, the between-healthcare system variance; Prediction Interval, the predicted effect size if we were to add a new healthcare system to the analysis. We excluded two adult healthcare systems (NUH and UKFR) from the meta-analysis due to low frequency of neurological diagnoses in their patient populations (< 1% of adult patients).
