# Supplementary material for: Neurological diagnoses in hospitalized COVID-19 patients associated with adverse outcomes: A multinational cohort study
Source: PLOS Digit Health. 2024 Apr 15;3(4):e0000484. doi: 10.1371/journal.pdig.0000484 (PMC11018281; doi:10.1371/journal.pdig.0000484)
Supplement: S3 Fig — Each grouped stacked bar chart represents the normalized distribution (%) of adults (A) or children (B) with a specific pre-admission health condition (that collectively constitute the Elixhauser Comorbidity Index) at each healthcare system for each neurological status during acute COVID-19 hospitalization. Neurological status included no neurological condition (NNC), central nervous system (CNS) diagnosis, and peripheral nervous system (PNS) diagnosis. Each stacked bar represents a healthcare system. With nk indicating the number of patients from healthcare system k for pre-admission health condition c, and n1, n2, and n3 indicating the number of patients from the NNC, PNS, and CNS group, respectively, we summed patients at each system as nk = n1+n2+n3. Missing bars indicate no patients for the given pre-admission health condition at a healthcare system (nk = n1 = n2 = n3 = 0). A bar with a single color indicates that patients with a given pre-admission health condition c at that healthcare system all had the same neurological status (e.g. nk = n1 or nk = n2 or nk = n3). (PDF) [file pdig.0000484.s003.pdf]

A. Adult

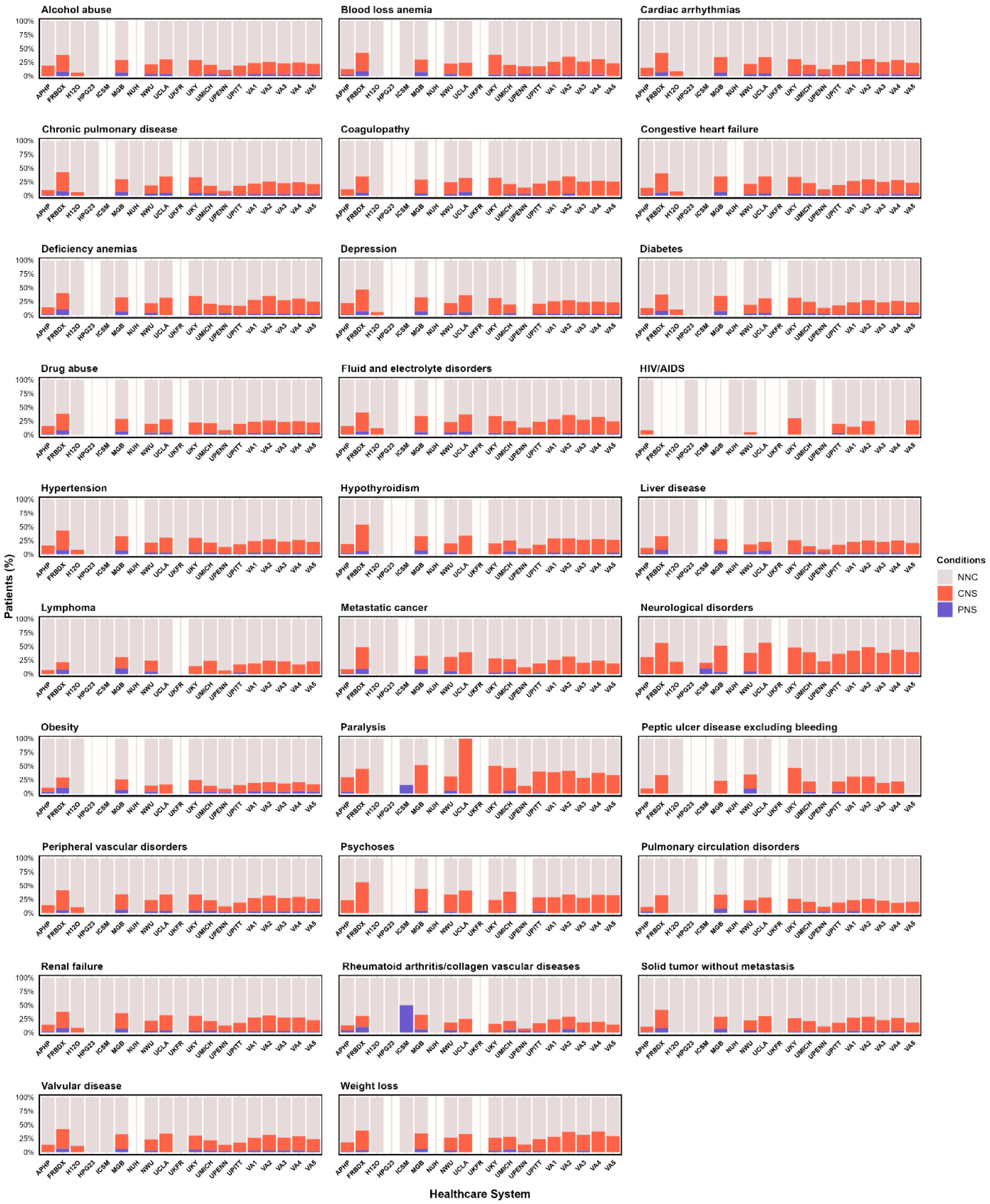

Alcohol abuse
Blood loss anemia
Cardiac arrhythmias
Chronic pulmonary disease
Coagulopathy
Congestive heart failure
Deficiency anemias
Depression
Diabetes
Drug abuse
Fluid and electrolyte disorders
HIV/AIDS
Hypertension
Hypothyroidism
Liver disease
Lymphoma
Metastatic cancer
Neurological disorders
Obesity
Paralysis
Peptic ulcer disease excluding bleeding
Peripheral vascular disorders
Psychoses
Pulmonary circulation disorders
Renal failure
Rheumatoid arthritis/collagen vascular diseases
Solid tumor without metastasis
Valvular disease
Weight loss
100%
75%
50%
25%
0%
APHP
FRBDX
H12O
HPG23
ICSM
MGB
NUH
NWU
UCLA
UKFR
UKY
UMICH
UPENN
UPITT
VA1
VA2
VA3
VA4
VA5
Patients (%)
Healthcare System
Conditions
NNC
CNS
PNS

B. Pediatric

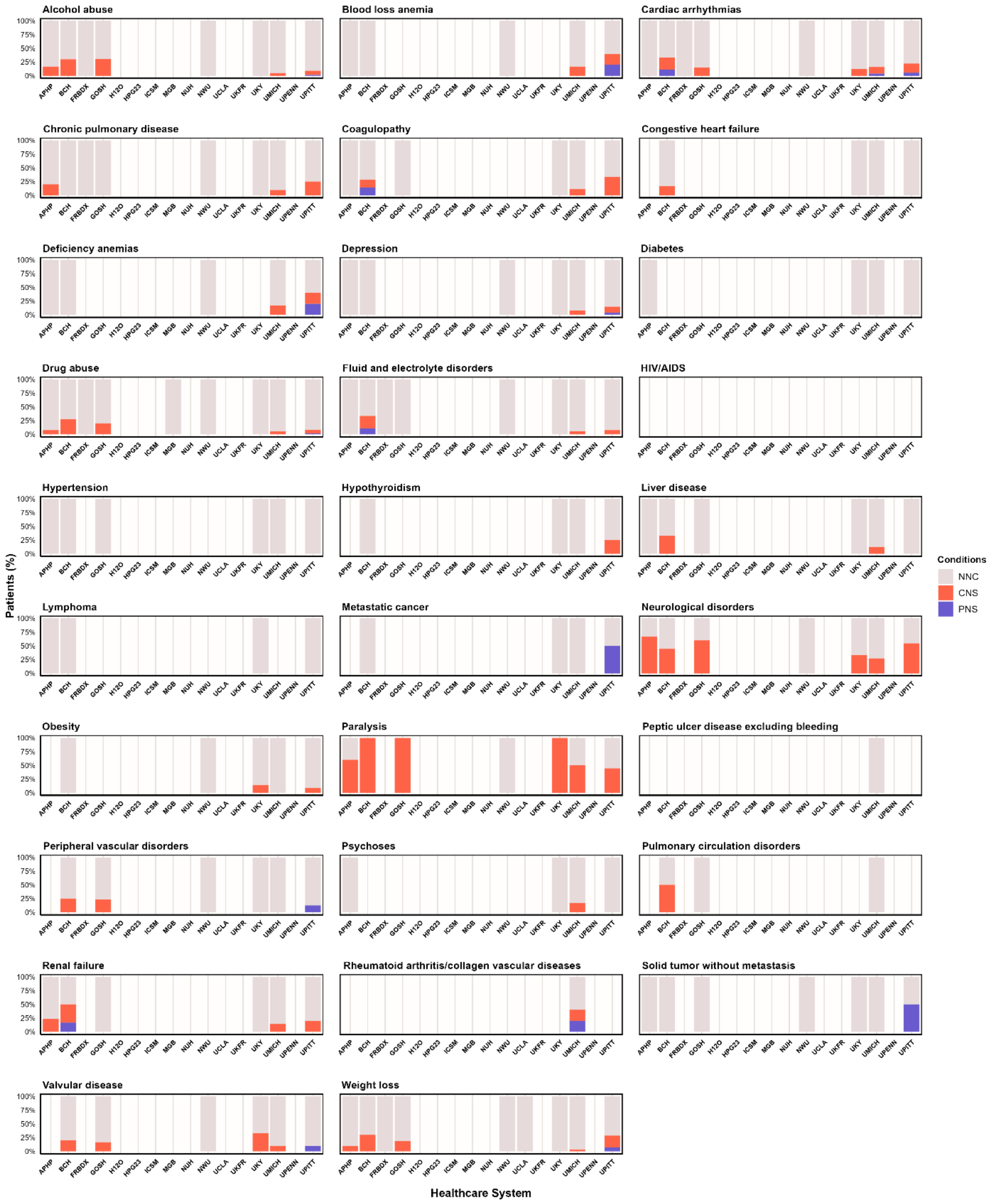

S3 Fig. Adult and pediatric pre-admission health conditions across healthcare systems. Each grouped stacked bar chart represents the normalized distribution (%) of adults (**A**) or children (**B**) with a specific pre-admission health condition (that collectively constitute the Elixhauser Comorbidity Index) at each healthcare system for each neurological status during acute COVID-19 hospitalization. Neurological status included no neurological condition (NNC), central nervous system (CNS) diagnosis, and peripheral nervous system (PNS) diagnosis. Each stacked bar represents a healthcare system. With nk indicating the number of patients from healthcare system k for pre-admission health condition c, and n1, n2, and n3 indicating the number of patients from the NNC, PNS, and CNS group, respectively, we summed patients at each system as nk = n1+n2+n3. Missing bars indicate no patients for the given pre-admission health condition at a healthcare system (nk=n1=n2=n3= 0). A bar with a single color indicates that patients with a given pre-admission health condition c at that healthcare system all had the same neurological status (e.g. nk=n1 or nk=n2 or nk=n3).
