# Supplementary material for: Neurological diagnoses in hospitalized COVID-19 patients associated with adverse outcomes: A multinational cohort study
Source: PLOS Digit Health. 2024 Apr 15;3(4):e0000484. doi: 10.1371/journal.pdig.0000484 (PMC11018281; doi:10.1371/journal.pdig.0000484)
Supplement: S4 Fig — Cox proportional hazard models were constructed using three censor cutoff periods (30, 60, and 90 days) and three methods of adjusting for pre-admission health conditions (i.e., pre-existing comorbidity burden): (1) the inclusion of the 29 individual covariates (i.e., health conditions) comprising the Elixhauser Comorbidity Index, (2) the Elixhauser summary score, and (3) the top 10 principal components computed from logistic principal component analysis (LPCA). Violin plots represent the distribution of concordance across healthcare systems (each black dot representing the concordance from a specific healthcare system). (PDF) [file pdig.0000484.s004.pdf]

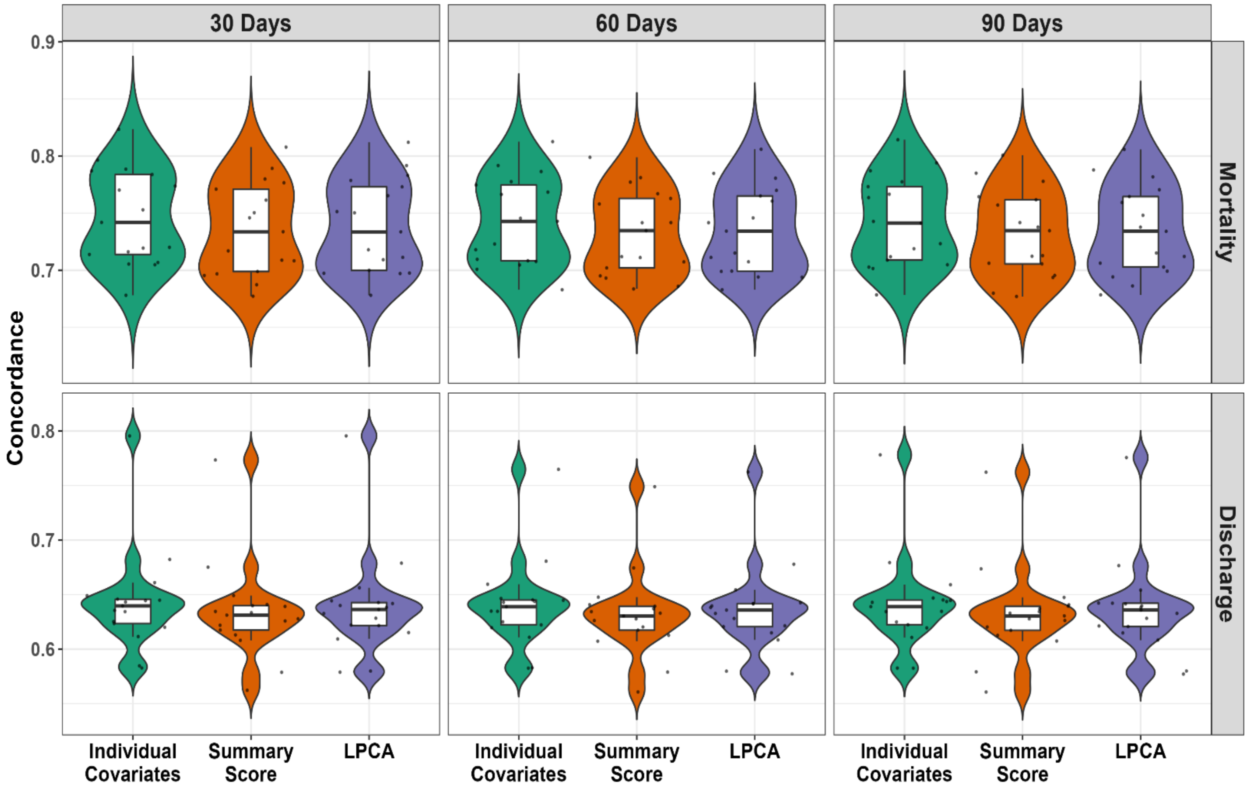


**S4 Fig. Concordance of Cox proportional hazard models.** Cox proportional hazard models were constructed using three censor cutoff periods (30, 60, and 90 days) and three methods of adjusting for pre-admission health conditions (*i.e.*, pre-existing comorbidity burden): (1) the inclusion of the 29 individual covariates (*i.e.*, health conditions) comprising the Elixhauser Comorbidity Index, (2) the Elixhauser summary score, and (3) the top 10 principal components computed from logistic principal component analysis (LPCA). Violin plots represent the distribution of concordance across healthcare systems (each black dot representing the concordance from a specific healthcare system).
