# Supplementary material for: Neurological diagnoses in hospitalized COVID-19 patients associated with adverse outcomes: A multinational cohort study
Source: PLOS Digit Health. 2024 Apr 15;3(4):e0000484. doi: 10.1371/journal.pdig.0000484 (PMC11018281; doi:10.1371/journal.pdig.0000484)
Supplement: S5 Fig — Cox proportional hazard models were constructed using three censor cutoff periods (30, 60, and 90 days) and three methods of adjusting for pre-admission health conditions (or comorbidity burden): (1) the inclusion of the 29 individual covariates (health conditions) comprising the Elixhauser Comorbidity Index, (2) the Elixhauser summary score, and (3) the top 10 principal components computed from logistic principal component analysis (LPCA). A random effects meta-analysis was performed to determine the pooled hazard ratio (HR) and 95% confidence intervals of each outcome. The meta-analysis was performed separately for central nervous system (CNS) and peripheral nervous system (PNS) diagnosis during acute COVID-19 hospitalization to evaluate the risk of each outcome with respect to patients with no neurological condition (NNC). (PDF) [file pdig.0000484.s005.pdf]

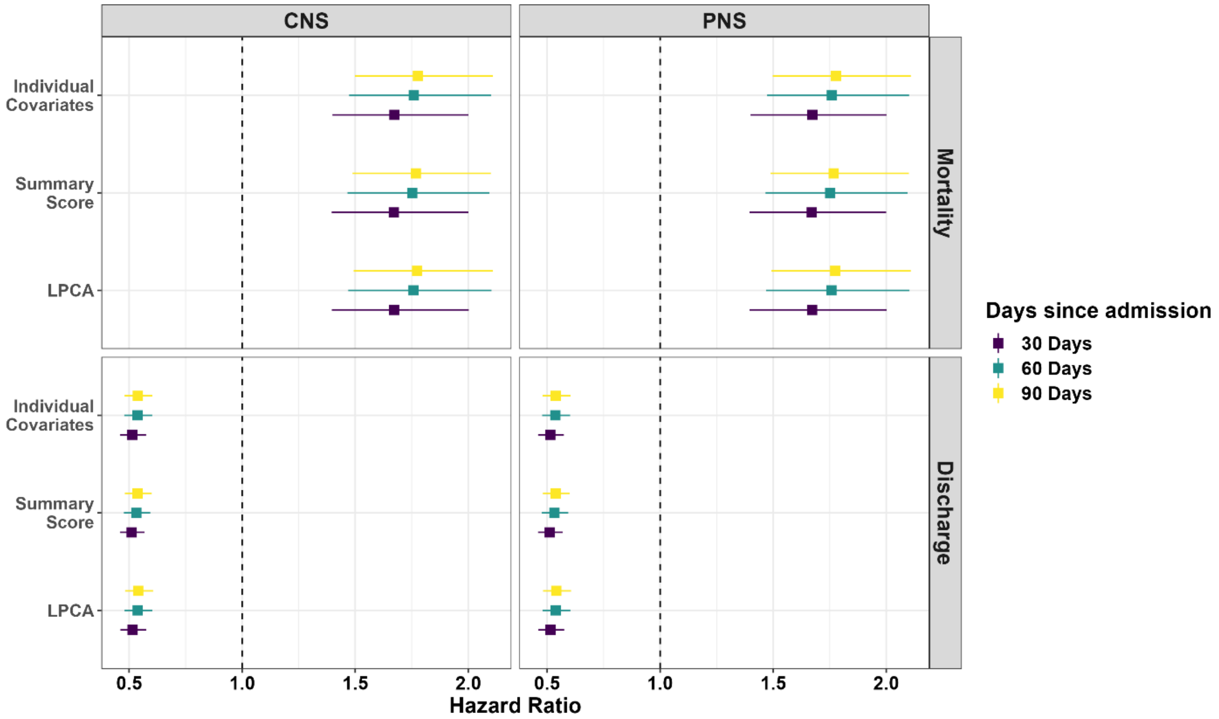

S5 Fig. Hazard ratios of a random effects meta-analysis of locally estimated Cox proportional hazard models. Cox proportional hazard models were constructed using three censor cutoff periods (30, 60, and 90 days) and three methods of adjusting for pre-admission health conditions (or comorbidity burden): (1) the inclusion of the 29 individual covariates (health conditions) comprising the Elixhauser Comorbidity Index, (2) the Elixhauser summary score, and (3) the top 10 principal components computed from logistic principal component analysis (LPCA). A random effects meta-analysis was performed to determine the pooled hazard ratio (HR) and 95% confidence intervals of each outcome. The meta-analysis was performed separately for central nervous system (CNS) and peripheral nervous system (PNS) diagnosis during acute COVID-19 hospitalization to evaluate the risk of each outcome with respect to patients with no neurological condition (NNC).
