# Supplementary material for: Neurological diagnoses in hospitalized COVID-19 patients associated with adverse outcomes: A multinational cohort study
Source: PLOS Digit Health. 2024 Apr 15;3(4):e0000484. doi: 10.1371/journal.pdig.0000484 (PMC11018281; doi:10.1371/journal.pdig.0000484)
Supplement: S6 Fig — For each outcome (hospital discharge, death), we conducted a random-effects meta-analysis to estimate the pooled hazard ratio and 95% confidence intervals for each age group using patients in the age group of 18–25 years as the reference group. The pooled hazard ratio for hospitalized patients with acute COVID-19 and concurrent central (CNS) and peripheral (PNS) nervous system diagnoses are also plotted as a reference. Dashed lines demarcate the hazard ratio at 1. (PDF) [file pdig.0000484.s006.pdf]

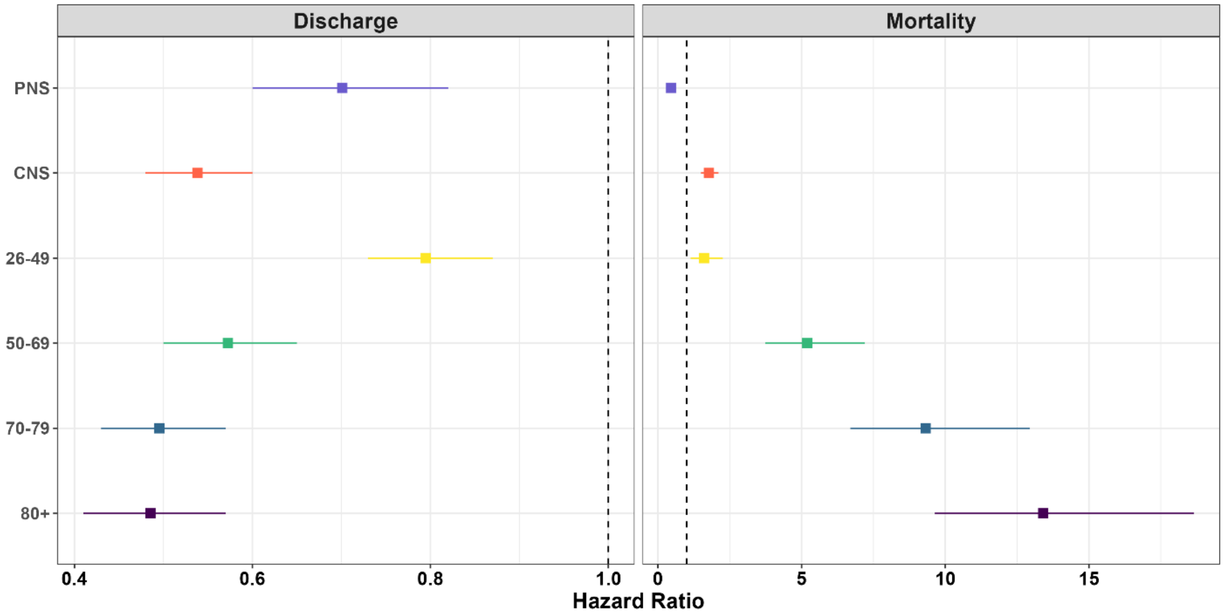

S6 Fig. Estimated hazard ratios by age group. For each outcome (hospital discharge, death), we conducted a random-effects meta-analysis to estimate the pooled hazard ratio and 95% confidence intervals for each age group using patients in the age group of 18-25 years as the reference group. The pooled hazard ratio for hospitalized patients with acute COVID-19 with concurrent peripheral (PNS) and central (CNS) nervous system diagnoses are also plotted as a reference. Dashed lines demarcate the hazard ratio at 1.
