# Supplementary material for: Neurological diagnoses in hospitalized COVID-19 patients associated with adverse outcomes: A multinational cohort study
Source: PLOS Digit Health. 2024 Apr 15;3(4):e0000484. doi: 10.1371/journal.pdig.0000484 (PMC11018281; doi:10.1371/journal.pdig.0000484)
Supplement: S1 Table — (PDF) [file pdig.0000484.s007.pdf]

S1 Table. Description of participating healthcare systems.

Healthcare System	Abbreviation	Country	Primary City	Hospitals within system	Adults included in the study	Children included in the study
Assistance Publique - Hôpitaux de Paris	APHP	France	Paris	39	25525	659
ASST Papa Giovanni XXIII Bergamo	HPG23	Italy	Bergamo	1	2758	125
Bordeaux University Hospital	FRBDX	France	Bordeaux	3	2237	113
Boston Children's Hospital	BCH	United States	Boston, MA	1	0	136
Great Ormond Street Hospital	GOSH	United Kingdom	London	1	0	112
Hospital Universitario 12 de Octubre	H12O	Spain	Madrid	1	5324	95
Istituti Clinici Scientifici Maugeri	ICSM	Italy	Pavia, Lumezzane/Brescia, Milan	3	459	1
Mass General Brigham	MGB	United States	Boston, MA	10	6474	73
Medical Center, University of Freiburg	UKRF	Germany	Freiburg	1	540	35
National University Hospital	NUH	Singapore	Singapore	1	268	23
Northwestern University	NWU	United States	Chicago, IL	10	6579	40
University of California, Los Angeles	UCLA	United States	Los Angeles, CA	2	1873	58
University of Kentucky	UKY	United States	Lexington, KY	3	1418	128
University of Michigan	UMICH	United States	Ann Arbor, MI	3	3520	353
University of Pennsylvania	UPENN	United States	Philadelphia, PA	5	8061	35
University of Pittsburgh / University of Pittsburgh Medical Center	UPITT	United States	Pittsburgh, PA	39	9911	212
Veterans Affairs Continental	VA4	United States	Continental	24	5518	0
Veterans Affairs Midwest	VA3	United States	Midwest	39	6568	0
Veterans Affairs North America	VA1	United States	North America	49	5512	0
Veterans Affairs Pacific	VA5	United States	Pacific	29	5084	0
Veterans Affairs Southwest	VA2	United States	Southwest	29	6402	0
**Total**				**293**	**104,031**	**2,198**
