# Supplementary material for: Neurological diagnoses in hospitalized COVID-19 patients associated with adverse outcomes: A multinational cohort study
Source: PLOS Digit Health. 2024 Apr 15;3(4):e0000484. doi: 10.1371/journal.pdig.0000484 (PMC11018281; doi:10.1371/journal.pdig.0000484)
Supplement: S2 Table — (PDF) [file pdig.0000484.s008.pdf]

S2 Table. Descriptions of the parent ICD-10 diagnosis codes for classifying concurrent central or peripheral nervous system manifestations during acute COVID-19 and their subcategory diagnosis codes.

Nervous System	Parent Category	Subcategory	ICD-10 Description	Abbreviated Description
Central	F29	NA	Unspecified psychosis not due to a substance or known physiological condition	Psychosis
Central	G03	NA	Meningitis due to other and unspecified causes	Meningitis
Central	G03	G03.0	Non-pyogenic meningitis	Meningitis
Central	G03	G03.1	Chronic meningitis	Meningitis
Central	G03	G03.2	Benign recurrent meningitis	Meningitis
Central	G03	G03.8	Meningitis due to other specified causes	Meningitis
Central	G03	G03.9	Meningitis, unspecified	Meningitis
Central	G04	NA	Encephalitis, myelitis and encephalomyelitis	Encephalitis, myelitis and encephalomyelitis
Central	G04	G04.0	Acute disseminated encephalitis and encephalomyelitis (ADEM)	Encephalitis, myelitis and encephalomyelitis
Central	G04	G04.1	Tropical spastic paraplegia	Encephalitis, myelitis and encephalomyelitis
Central	G04	G04.2	Bacterial meningoencephalitis and meningomyelitis, not elsewhere classified	Encephalitis, myelitis and encephalomyelitis
Central	G04	G04.3	Acute necrotizing hemorrhagic encephalopathy	Encephalitis, myelitis and encephalomyelitis
Central	G04	G04.8	Other encephalitis, myelitis and encephalomyelitis	Encephalitis, myelitis and encephalomyelitis
Central	G04	G04.9	Encephalitis, myelitis and encephalomyelitis, unspecified	Encephalitis, myelitis and encephalomyelitis
Central	G40	NA	Epilepsy and recurrent seizures	Epilepsy and recurrent seizures
Central	G40	G40.0	Focal partial idiopathic epilepsy and epileptic syndromes with seizures of localized onset	Epilepsy and recurrent seizures
Central	G40	G40.1	Focal partial symptomatic epilepsy and epileptic syndromes with simple partial seizures	Epilepsy and recurrent seizures
Central	G40	G40.2	Focal partial symptomatic epilepsy and epileptic syndromes with complex partial seizures	Epilepsy and recurrent seizures
Central	G40	G40.3	Generalized idiopathic epilepsy and epileptic syndromes	Epilepsy and recurrent seizures
Central	G40	G40.A	Absence epileptic syndrome	Epilepsy and recurrent seizures
Central	G40	G40.B	Juvenile myoclonic epilepsy	Epilepsy and recurrent seizures
Central	G40	G40.4	Other generalized epilepsy and epileptic syndromes	Epilepsy and recurrent seizures
Central	G40	G40.5	Epileptic seizures related to external causes	Epilepsy and recurrent seizures
Central	G40	G40.8	Other epilepsy and recurrent seizures	Epilepsy and recurrent seizures
Central	G40	G40.9	Epilepsy, unspecified	Epilepsy and recurrent seizures
Central	G44	NA	Other headache syndromes	Headache syndromes
Central	G44	G44.0	Cluster headaches and other trigeminal autonomic cephalgias (TAC)	Headache syndromes
Central	G45	G44.1	Vascular headache, not elsewhere classified	Headache syndromes
Central	G46	G44.2	Tension type headache	Headache syndromes
Central	G47	G44.3	Post traumatic headache	Headache syndromes
Central	G48	G44.4	Drug induced headache, not elsewhere classified	Headache syndromes

Central	G49	G44.5	Complicated headache syndromes	Headache syndromes
Central	G50	G44.8	Other specified headache syndromes	Headache syndromes
Central	G45	NA	Transient cerebral ischemic attacks and related syndromes	Transient cerebral ischemic attacks and related syndromes
Central	G45	G45.0	Vertebro-basilar artery syndrome	Transient cerebral ischemic attacks and related syndromes
Central	G45	G45.1	Carotid artery syndrome (hemispheric)	Transient cerebral ischemic attacks and related syndromes
Central	G45	G45.2	Multiple and bilateral precerebral artery syndromes	Transient cerebral ischemic attacks and related syndromes
Central	G45	G45.3	Amaurosis fugax	Transient cerebral ischemic attacks and related syndromes
Central	G45	G45.4	Transient global amnesia	Transient cerebral ischemic attacks and related syndromes
Central	G45	G45.8	Other transient cerebral ischemic attacks and related syndromes	Transient cerebral ischemic attacks and related syndromes
Central	G45	G45.9	Transient cerebral ischemic attack, unspecified	Transient cerebral ischemic attacks and related syndromes
Central	G46	NA	Vascular syndromes of brain in cerebrovascular disease	Vascular syndromes of brain in cerebrovascular disease
Central	G46	G46.0	Middle cerebral artery syndrome	Vascular syndromes of brain in cerebrovascular disease
Central	G46	G46.1	Anterior cerebral artery syndrome	Vascular syndromes of brain in cerebrovascular disease
Central	G46	G46.2	Posterior cerebral artery syndrome	Vascular syndromes of brain in cerebrovascular disease
Central	G46	G46.3	Brainstem stroke syndrome	Vascular syndromes of brain in cerebrovascular disease
Central	G46	G46.4	Cerebellar stroke syndrome	Vascular syndromes of brain in cerebrovascular disease
Central	G46	G46.5	Pure motor lacunar syndrome	Vascular syndromes of brain in cerebrovascular disease
Central	G46	G46.6	Pure sensory lacunar syndrome	Vascular syndromes of brain in cerebrovascular disease
Central	G46	G46.7	Other lacunar syndromes	Vascular syndromes of brain in cerebrovascular disease
Central	G46	G46.8	Other vascular syndromes of brain in cerebrovascular diseases	Vascular syndromes of brain in cerebrovascular disease
Peripheral	G61	NA	Inflammatory polyneuropathy	Inflammatory polyneuropathy
Peripheral	G61	G61.0	Guillain Barre syndrome	Inflammatory polyneuropathy
Peripheral	G61	G61.1	Serum neuropathy	Inflammatory polyneuropathy
Peripheral	G61	G61.8	Other inflammatory polyneuropathies	Inflammatory polyneuropathy
Peripheral	G61	G61.9	Inflammatory polyneuropathy, unspecified	Inflammatory polyneuropathy
Peripheral	G65	NA	Sequelae of inflammatory and toxic polyneuropathies	Inflammatory and toxic polyneuropathies sequelae
Peripheral	G65	G65.0	Sequelae of Guillain Barre syndrome	Inflammatory and toxic polyneuropathies sequelae
Peripheral	G65	G65.1	Sequelae of other inflammatory polyneuropathy	Inflammatory and toxic polyneuropathies sequelae
Peripheral	G65	G65.2	Sequelae of toxic polyneuropathy	Inflammatory and toxic polyneuropathies sequelae
Peripheral	G72	NA	Other and unspecified myopathies	Myopathies
Peripheral	G72	G72.0	Drug induced myopathy	Myopathies
Peripheral	G72	G72.1	Alcoholic myopathy	Myopathies
Peripheral	G72	G72.2	Myopathy due to other toxic agents	Myopathies
Peripheral	G72	G72.3	Periodic paralysis	Myopathies
Peripheral	G72	G72.4	Inflammatory and immune myopathies, not elsewhere classified	Myopathies
Peripheral	G72	G72.8	Other specified myopathies	Myopathies

Peripheral	G72	G72.9	Myopathy, unspecified	Myopathies
Central	G93	G93	Other disorders of the brain	Disorders of the brain
Central	G93	G93.0	Cerebral cysts	Disorders of the brain
Central	G93	G93.1	Anoxic brain damage, not elsewhere classified	Disorders of the brain
Central	G93	G93.2	Benign intracranial hypertension	Disorders of the brain
Central	G93	G93.3	Post-viral fatigue syndrome	Disorders of the brain
Central	G93	G93.4	Other and unspecified encephalopathy	Disorders of the brain
Central	G93	G93.5	Compression of brain	Disorders of the brain
Central	G93	G93.6	Cerebral edema	Disorders of the brain
Central	G93	G93.7	Reye's syndrome	Disorders of the brain
Central	G93	G93.8	Other specified disorders of brain	Disorders of the brain
Central	G93	G93.9	Disorders of brain, unspecified	Disorders of the brain
Central	H54	NA	Blindness and low vision	Blindness and low vision
Central	H54	H54.0	Blindness, both eyes	Blindness and low vision
Central	H54	H54.1	Blindness, one eye, low vision other eye	Blindness and low vision
Central	H54	H54.2	Low vision, both eyes	Blindness and low vision
Central	H54	H54.3	Unqualified visual loss, both eyes	Blindness and low vision
Central	H54	H54.4	Blindness, one eye	Blindness and low vision
Central	H54	H54.5	Low vision, one eye	Blindness and low vision
Central	H54	H54.6	Unqualified visual loss, one eye	Blindness and low vision
Central	H54	H54.7	Unspecified visual loss	Blindness and low vision
Central	H54	H54.8	Legal blindness, as defined in USA	Blindness and low vision
Central	I60	NA	Nontraumatic subarachnoid hemorrhage	Subarachnoid hemorrhage (non-traumatic)
Central	I60	I60.0	Nontraumatic subarachnoid hemorrhage from carotid siphon and bifurcation	Subarachnoid hemorrhage (non-traumatic)
Central	I60	I60.1	Nontraumatic subarachnoid hemorrhage from middle cerebral artery	Subarachnoid hemorrhage (non-traumatic)
Central	I60	I60.2	Nontraumatic subarachnoid hemorrhage from anterior communicating artery	Subarachnoid hemorrhage (non-traumatic)
Central	I60	I60.3	Nontraumatic subarachnoid hemorrhage from posterior communicating artery	Subarachnoid hemorrhage (non-traumatic)
Central	I60	I60.4	Nontraumatic subarachnoid hemorrhage from basilar artery	Subarachnoid hemorrhage (non-traumatic)
Central	I60	I60.5	Nontraumatic subarachnoid hemorrhage from vertebral artery	Subarachnoid hemorrhage (non-traumatic)
Central	I60	I60.6	Nontraumatic subarachnoid hemorrhage from other intracranial arteries	Subarachnoid hemorrhage (non-traumatic)
Central	I60	I60.7	Nontraumatic subarachnoid hemorrhage from unspecified intracranial artery	Subarachnoid hemorrhage (non-traumatic)
Central	I60	I60.8	Other nontraumatic subarachnoid hemorrhage	Subarachnoid hemorrhage (non-traumatic)
Central	I60	I60.9	Nontraumatic subarachnoid hemorrhage, unspecified	Subarachnoid hemorrhage (non-traumatic)
Central	I61	NA	Nontraumatic intracerebral hemorrhage	Intracerebral hemorrhage (non-traumatic)
Central	161	I61.0	Nontraumatic intracranial hemorrhage in hemisphere, subcortical	Intracerebral hemorrhage (non-traumatic)
Central	161	I61.1	Nontraumatic intracerebral hemorrhage in hemisphere, cortical	Intracerebral hemorrhage (non-traumatic)
Central	161	I61.2	Nontraumatic intracerebral hemorrhage in hemisphere, unspecified	Intracerebral hemorrhage (non-traumatic)
Central	161	I61.3	Nontraumatic intracranial hemorrhage in brain stem	Intracerebral hemorrhage (non-traumatic)

Central	161	I61.4	Nontraumatic intracranial hemorrhage in cerebellum	Intracerebral hemorrhage (non-traumatic)
Central	161	I61.5	Nontraumatic intracranial hemorrhage, intraventricular	Intracerebral hemorrhage (non-traumatic)
Central	161	I61.6	Nontraumatic intracranial hemorrhage, multiple localized	Intracerebral hemorrhage (non-traumatic)
Central	161	I61.8	Other nontraumatic intracerebral hemorrhage	Intracerebral hemorrhage (non-traumatic)
Central	161	I61.9	Nontraumatic intracerebral hemorrhage, unspecified	Intracerebral hemorrhage (non-traumatic)
Central	I62	NA	Other and unspecified nontraumatic intracranial hemorrhage	Intracranial hemorrhage (non-traumatic)
Central	I62	I62.0	Nontraumatic subdural hemorrhage	Intracranial hemorrhage (non-traumatic)
Central	I62	I62.1	Nontraumatic extradural hemorrhage	Intracranial hemorrhage (non-traumatic)
Central	I62	I62.9	Nontraumatic intracranial hemorrhage, unspecified	Intracranial hemorrhage (non-traumatic)
Central	I67	NA	Other cerebrovascular diseases	Cerebrovascular diseases
Central	I67	I67.0	Dissection of cerebral arteries, non-ruptured	Cerebrovascular diseases
Central	I67	I67.1	Cerebral aneurysm, non-ruptured	Cerebrovascular diseases
Central	I67	I67.2	Cerebral atherosclerosis	Cerebrovascular diseases
Central	I67	I67.3	Progressive vascular leukoencephalopathy	Cerebrovascular diseases
Central	I67	I67.4	Hypertensive encephalopathy	Cerebrovascular diseases
Central	I67	I67.5	Moyamoya disease	Cerebrovascular diseases
Central	I67	I67.6	Non-pyogenic thrombosis of intracranial venous system	Cerebrovascular diseases
Central	I67	I67.7	Cerebral arteritis, not elsewhere specified	Cerebrovascular diseases
Central	I67	I67.8	Other specified cerebrovascular diseases	Cerebrovascular diseases
Central	I67	I67.9	Cerebrovascular disease, unspecified	Cerebrovascular diseases
Peripheral	M60	NA	Myositis	Myositis
Peripheral	M60	M60.0	Infective myositis	Myositis
Peripheral	M60	M60.1	Interstitial myositis	Myositis
Peripheral	M60	M60.2	Foreign body granuloma of soft tissue, not elsewhere classified	Myositis
Peripheral	M60	M60.8	Other myositis	Myositis
Peripheral	M60	M60.9	Myositis, unspecified	Myositis
Central	R27	NA	Other lack of coordination	Lack of coordination
Central	R27	R27.0	Ataxia, unspecified	Lack of coordination
Central	R27	R27.8	Other lack of coordination	Lack of coordination
Central	R27	R27.9	Unspecified lack of coordination	Lack of coordination
Central	R41	NA	Other symptoms and signs involving cognitive functions and awareness	Cognitive functions and awareness
Central	R41	R41.0	Disorientation, unspecified	Cognitive functions and awareness
Central	R41	R41.1	Anterograde amnesia	Cognitive functions and awareness
Central	R41	R41.2	Retrograde amnesia	Cognitive functions and awareness
Central	R41	R41.3	Other amnesia	Cognitive functions and awareness
Central	R41	R41.4	Neurologic neglect syndrome	Cognitive functions and awareness
Central	R41	R41.8	Other symptoms and signs involving cognitive functions and awareness	Cognitive functions and awareness
Central	R41	R41.9	Unspecified symptoms and signs involving cognitive functions and awareness	Cognitive functions and awareness

Peripheral	R42	NA	Dizziness and giddiness	Dizziness and giddiness
Peripheral	R43	NA	Disturbances of smell and taste	Disturbances of smell and taste
Peripheral	R43	R43.0	Anosmia	Disturbances of smell and taste
Peripheral	R43	R43.1	Parosmia	Disturbances of smell and taste
Peripheral	R43	R43.2	Parageusia	Disturbances of smell and taste
Peripheral	R43	R43.8	Other disturbances of smell and taste	Disturbances of smell and taste
Peripheral	R43	R43.9	Unspecified disturbances of smell and taste	Disturbances of smell and taste
