# Supplementary material for: Neurological diagnoses in hospitalized COVID-19 patients associated with adverse outcomes: A multinational cohort study
Source: PLOS Digit Health. 2024 Apr 15;3(4):e0000484. doi: 10.1371/journal.pdig.0000484 (PMC11018281; doi:10.1371/journal.pdig.0000484)
Supplement: S3 Table — Notes: 1. Complicated and uncomplicated diabetes were combined as one condition. Likewise, complicated and uncomplicated hypertension were combined as one condition. (PDF) [file pdig.0000484.s009.pdf]

S3 Table. List of the parent category ICD-10 diagnosis codes mapped to each condition of the Elixhauser Comorbidity Index and its associated weight.

Comorbidity	ICD-10 Codes	Van Walraven Weight
Alcohol abuse	E52, F10, G62, I42, K29, K70, T51, Z50, Z71, Z72	0
Blood loss anemia	D50	-2
Cardiac arrhythmias	I44, I45, I47, I48, I49, R00, T82, Z45, Z95	5
Chronic pulmonary disease	I27, J40, J41, J42, J43, J44, J45, J46, J47, J60, J61, J62, J63, J64, J65, J66, J67, J68, J70	3
Coagulopathy	D65, D66, D67, D68, D69	3
Congestive heart failure	I09, I11, I13, I25, I42, I43, I50, P29	7
Deficiency anemias	D50, D51, D52, D53	-2
Depression	F20, F31, F32, F33, F34, F41, F43	-3
Diabetes [1]	E10, E11, E12, E13, E14, E10, E11, E12, E13, E14	0
Drug abuse	F11, F12, F13, F14, F15, F16, F18, F19, Z71, Z72	-7
Fluid and electrolyte disorders	E22, E86, E87	5
HIV/AIDS	B20, B21, B22, B24	0
Hypertension [1]	I10, I11, I12, I13, I15	0
Hypothyroidism	E00, E01, E02, E03, E89	0
Liver disease	B18, I85, I86, I98, K70, K71, K72, K73, K74, K76, Z94	11
Lymphoma	C81, C82, C83, C84, C85, C88, C90, C96	9
Metastatic cancer	C77, C78, C79, C80	12
Obesity	E66	-4
Neurological disorders	G10, G11, G12, G13, G20, G21, G22, G25, G31, G32, G35, G36, G37, G40, G41, G93, R47, R56	6
Paralysis	G04, G11, G80, G81, G82, G83	7
Peptic ulcer disease excluding bleeding	K25, K26, K27, K28	0
Peripheral vascular disorders	I70, I71, I73, I77, I79, K55, Z95	2
Psychoses	F20, F22, F23, F24, F25, F28, F29, F30, F31	0
Pulmonary circulation disorders	I26, I27, I28	4
Renal failure	I12, I13, N18, N19, N25, Z49, Z94, Z99	5
Rheumatoid arthritis/collagen vascular diseases	L94, M05, M06, M08, M12, M30, M31, M32, M33, M34, M35, M45, M46	0

Solid tumor without metastasis	C00, C01, C02, C03, C04, C05, C06, C07, C08, C09, C10, C11, C12, C13, C14, C15, C16, C17, C18, C19, C20, C21, C22, C23, C24, C25, C26, C30, C31, C32, C33, C34, C37, C38, C39, C40, C41, C43, C45, C46, C47, C48, C49, C50, C51, C52, C53, C54, C55, C56, C57, C58, C60, C61, C62, C63, C64, C65, C66, C67, C68, C69, C70, C71, C72, C73, C74, C75, C76, C97	4
Valvular disease	A52, I05, I06, I07, I08, I09, I34, I35, I36, I37, I38, I39, Q23, Z95	-1
Weight loss	E40, E41, E42, E43, E44, E45, E46, R63, R64	6

Notes:

1. Complicated and uncomplicated diabetes were combined as one condition. Likewise, complicated and uncomplicated hypertension were combined as one condition.
