# Supplementary material for: Neurological diagnoses in hospitalized COVID-19 patients associated with adverse outcomes: A multinational cohort study
Source: PLOS Digit Health. 2024 Apr 15;3(4):e0000484. doi: 10.1371/journal.pdig.0000484 (PMC11018281; doi:10.1371/journal.pdig.0000484)
Supplement: S4 Table — Notes: 1. Complicated and uncomplicated diabetes were combined as one condition. Likewise, complicated and uncomplicated hypertension were combined as one condition. 2. ICD-10 descriptions were curated using the icd R package [1]. Supplemental Citation 1. Wasey JO, Frank SM, Rehman MA. icd: Efficient Computation of Comorbidities from ICD Codes Using Sparse Matrix Multiplication in R. Journal of Statistical Software. 2018. Available: https://jackwasey.github.io/icd/articles/efficiency-prebuilt.pdf (PDF) [file pdig.0000484.s010.pdf]

S4 Table. Descriptions of the parent category ICD-10 diagnosis codes mapped to each condition of the Elixhauser Comorbidity Index.

Comorbidity	ICD-10 Definition
**Alcohol abuse**	E52 - Niacin deficiency [pellagra]<br>F10 - Alcohol related disorders<br>G62 - Other and unspecified polyneuropathies<br>I42 - Cardiomyopathy<br>K29 - Gastritis and duodenitis<br>K70 - Alcoholic liver disease<br>T51 - Toxic effect of alcohol<br>Z71 - Persons encountering health services for other counseling and medical advice, not elsewhere classified<br>Z72 - Problems related to lifestyle
**Blood loss anemia**	D50 - Iron deficiency anemia
**Cardiac arrhythmias**	I44 - Atrioventricular and left bundle-branch block<br>I45 - Other conduction disorders<br>I47 - Paroxysmal tachycardia<br>I48 - Atrial fibrillation and flutter<br>I49 - Other cardiac arrhythmias<br>R00 - Abnormalities of heart beat<br>T82 - Complications of cardiac or vascular prosthetic devices, implants and grafts<br>Z45 - Encounter for adjustment and management of implanted device<br>Z95 - Presence of cardiac and vascular implants and grafts
**Chronic pulmonary disease**	I27 - Other pulmonary heart diseases<br>J40 - Bronchitis, not specified as acute or chronic<br>J41 - Simple and mucopurulent chronic bronchitis<br>J42 - Unspecified chronic bronchitis<br>J43 - Emphysema<br>J44 - Other chronic obstructive pulmonary disease<br>J45 - Asthma<br>J47 - Bronchiectasis<br>J60 – Coal worker's pneumoconiosis<br>J61 - Pneumoconiosis due to asbestos and other mineral fibers<br>J62 - Pneumoconiosis due to dust containing silica<br>J63 - Pneumoconiosis due to other inorganic dusts<br>J64 - Unspecified pneumoconiosis<br>J65 - Pneumoconiosis associated with tuberculosis<br>J66 - Airway disease due to specific organic dust<br>J67 - Hypersensitivity pneumonitis due to organic dust<br>J68 - Respiratory conditions due to inhalation of chemicals, gases, fumes and vapors<br>J70 - Respiratory conditions due to other external agents
**Coagulopathy**	D65 - Disseminated intravascular coagulation<br>D66 - Hereditary factor VIII deficiency<br>D67 - Hereditary factor IX deficiency<br>D68 - Other coagulation defects<br>D69 - Purpura and other hemorrhagic conditions
**Congestive heart failure**	I09 - Other rheumatic heart diseases<br>I11 - Hypertensive heart disease<br>I13 - Hypertensive heart and chronic kidney disease<br>I25 - Chronic ischemic heart disease<br>I42 - Cardiomyopathy<br>I43 - Cardiomyopathy in diseases classified elsewhere<br>I50 - Heart failure<br>P29 - Cardiovascular disorders originating in the perinatal period
**Deficiency anemias**	D50 - Iron deficiency anemia<br>D51 - Vitamin B12 deficiency anemia<br>D52 - Folate deficiency anemia<br>D53 - Other nutritional anemias
**Depression**	F20 - Schizophrenia<br>F31 - Bipolar disorder<br>F32 - Major depressive disorder, single episode<br>F33 - Major depressive disorder, recurrent<br>F34 - Persistent mood [affective] disorders<br>F41 - Other anxiety disorders<br>F43 - Reaction to severe stress, and adjustment disorders

**Diabetes**[1]	E10 - Type 1 diabetes mellitus<br>E11 - Type 2 diabetes mellitus<br>E13 - Other specified diabetes mellitus
**Drug abuse**	F11 - Opioid related disorders<br>F12 - Cannabis related disorders<br>F13 - Sedative, hypnotic, or anxiolytic related disorders<br>F14 - Cocaine related disorders<br>F15 - Other stimulant related disorders<br>F16 - Hallucinogen related disorders<br>F18 - Inhalant related disorders<br>F19 - Other psychoactive substance related disorders<br>Z71 - Persons encountering health services for other counseling and medical advice, not elsewhere classified<br>Z72 - Problems related to lifestyle
**Fluid and electrolyte disorders**	E22 - Hyperfunction of pituitary gland<br>E86 - Volume depletion<br>E87 - Other disorders of fluid, electrolyte and acid-base balance
**HIV / AIDS**	B20 - Human immunodeficiency virus [HIV] disease
**Hypertension**[1]	I10 - Essential (primary) hypertension
**Hypothyroidism**	E00 - Congenital iodine-deficiency syndrome<br>E01 - Iodine-deficiency related thyroid disorders and allied conditions<br>E02 - Subclinical iodine-deficiency hypothyroidism<br>E03 - Other hypothyroidism<br>E89 - Postprocedural endocrine and metabolic complications and disorders, not elsewhere classified
**Liver disease**	B18 - Chronic viral hepatitis<br>I85 - Esophageal varices<br>I86 - Varicose veins of other sites<br>K70 - Alcoholic liver disease<br>K71 - Toxic liver disease<br>K72 - Hepatic failure, not elsewhere classified<br>K73 - Chronic hepatitis, not elsewhere classified<br>K74 - Fibrosis and cirrhosis of liver<br>K76 - Other diseases of liver<br>Z94 - Transplanted organ and tissue status
**Lymphoma**	C81 - Hodgkin lymphoma<br>C82 - Follicular lymphoma<br>C83 - Non-follicular lymphoma<br>C84 - Mature T/NK-cell lymphomas<br>C85 - Other and unspecified types of non-Hodgkin lymphoma<br>C88 - Malignant immunoproliferative diseases and certain other B-cell lymphomas<br>C90 - Multiple myeloma and malignant plasma cell neoplasms<br>C96 - Other and unspecified malignant neoplasms of lymphoid, hematopoietic and related tissue
**Metastatic cancer**	C77 - Secondary malignant neoplasm of other and unspecified sites<br>C78 - Malignant neuroendocrine tumors<br>C78 - Secondary malignant neoplasm of other and unspecified sites<br>C79 - Malignant neuroendocrine tumors<br>C79 - Secondary neuroendocrine tumors<br>C80 - Malignant neoplasm without specification of site
**Neurological disorders**	G10 - Huntington's disease<br>G11 - Hereditary ataxia<br>G12 - Spinal muscular atrophy and related syndromes<br>G13 - Systemic atrophies primarily affecting central nervous system in diseases classified elsewhere<br>G20 - Parkinson's disease<br>G21 - Secondary parkinsonism<br>G25 - Other extrapyramidal and movement disorders<br>G31 - Other degenerative diseases of nervous system, not elsewhere classified<br>G32 - Other degenerative disorders of nervous system in diseases classified elsewhere<br>G35 - Multiple sclerosis<br>G36 - Other acute disseminated demyelination<br>G37 - Other demyelinating diseases of central nervous system<br>G40 - Epilepsy and recurrent seizures<br>G93 - Other disorders of brain<br>R47 - Speech disturbances, not elsewhere classified<br>R56 - Convulsions, not elsewhere classified
**Obesity**	E66 - Overweight and obesity

**Paralysis**	G04 - Encephalitis, myelitis and encephalomyelitis<br>G11 - Hereditary ataxia<br>G80 - Cerebral palsy<br>G81 - Hemiplegia and hemiparesis<br>G82 - Paraplegia (paraparesis) and quadriplegia (quadriparesis)<br>G83 - Other paralytic syndromes
**Peptic ulcer disease excluding bleeding**	K25 - Gastric ulcer<br>K26 - Duodenal ulcer<br>K27 - Peptic ulcer, site unspecified<br>K28 - Gastrojejunal ulcer
**Peripheral vascular disorders**	I70 - Atherosclerosis<br>I71 - Aortic aneurysm and dissection<br>I73 - Other peripheral vascular diseases<br>I77 - Other disorders of arteries and arterioles<br>I79 - Disorder of arteries, arterioles and capillaries in diseases classified elsewhere<br>K55 - Vascular disorders of intestine<br>Z95 - Presence of cardiac and vascular implants and grafts
**Psychoses**	F20 - Schizophrenia<br>F22 - Delusional disorders<br>F23 - Brief psychotic disorder<br>F24 - Shared psychotic disorder<br>F25 - Schizoaffective disorders<br>F28 - Other psychotic disorder not due to a substance or known physiological condition<br>F29 - Unspecified psychosis not due to a substance or known physiological condition<br>F30 - Manic episode<br>F31 - Bipolar disorder
**Pulmonary circulation disorders**	I26 - Pulmonary embolism<br>I27 - Other pulmonary heart diseases<br>I28 - Other diseases of pulmonary vessels
**Renal failure**	I12 - Hypertensive chronic kidney disease<br>I13 - Hypertensive heart and chronic kidney disease<br>N18 - Chronic kidney disease (CKD)<br>N19 - Unspecified kidney failure<br>N25 - Disorders resulting from impaired renal tubular function<br>Z49 - Encounter for care involving renal dialysis<br>Z94 - Transplanted organ and tissue status<br>Z99 - Dependence on enabling machines and devices, not elsewhere classified
**Rheumatoid arthritis / collagen vascular diseases**	L94 - Other localized connective tissue disorders<br>M05 - Rheumatoid arthritis with rheumatoid factor<br>M06 - Other rheumatoid arthritis<br>M08 - Juvenile arthritis<br>M12 - Chronic gout<br>M12 - Osteoarthritis of first carpometacarpal joint<br>M12 - Other and unspecified osteoarthritis<br>M30 - Polyarteritis nodosa and related conditions<br>M31 - Other necrotizing vasculopathies<br>M32 - Systemic lupus erythematosus (SLE)<br>M33 - Dermatopolymyositis<br>M34 - Systemic sclerosis [scleroderma]<br>M35 - Other systemic involvement of connective tissue<br>M45 - Ankylosing spondylitis<br>M46 - Other inflammatory spondylopathies
**Solid tumor without metastasis**	C00 - Malignant neoplasm of lip<br>C01 - Malignant neoplasm of base of tongue<br>C02 - Malignant neoplasm of other and unspecified parts of tongue<br>C03 - Malignant neoplasm of gum<br>C04 - Malignant neoplasm of floor of mouth<br>C05 - Malignant neoplasm of palate<br>C06 - Malignant neoplasm of other and unspecified parts of mouth<br>C07 - Malignant neoplasm of parotid gland<br>C08 - Malignant neoplasm of other and unspecified major salivary glands<br>C09 - Malignant neoplasm of tonsil<br>C10 - Malignant neoplasm of oropharynx<br>C11 - Malignant neoplasm of nasopharynx<br>C12 - Malignant neoplasm of pyriform sinus<br>C13 - Malignant neoplasm of hypopharynx<br>C14 - Malignant neoplasm of sites in the lip, oral cavity and pharynx<br>C15 - Malignant neoplasm of esophagus<br>C16 - Malignant neoplasm of stomach

	C17 - Malignant neoplasm of small intestine<br>C18 - Malignant neoplasm of colon<br>C19 - Malignant neoplasm of rectosigmoid junction<br>C20 - Malignant neoplasm of rectum<br>C21 - Malignant neoplasm of anus and anal canal<br>C22 - Malignant neoplasm of liver and intrahepatic bile ducts<br>C23 - Malignant neoplasm of gallbladder<br>C24 - Malignant neoplasm of other and unspecified parts of biliary tract<br>C25 - Malignant neoplasm of pancreas<br>C26 - Malignant neoplasm of other and ill-defined digestive organs<br>C30 - Malignant neoplasm of nasal cavity and middle ear<br>C31 - Malignant neoplasm of accessory sinuses<br>C32 - Malignant neoplasm of larynx<br>C33 - Malignant neoplasm of trachea<br>C34 - Malignant neoplasm of bronchus and lung<br>C37 - Malignant neoplasm of thymus<br>C38 - Malignant neoplasm of heart, mediastinum and pleura<br>C39 - Malignant neoplasm of sites in the resp sys and intrathoracic organs<br>C40 - Malignant neoplasm of bone and articular cartilage of limbs<br>C41 - Malignant neoplasm of bone and articular cartilage of and unspecified sites<br>C43 - Malignant melanoma of skin<br>C45 - Malignant neoplasm of peripheral nerves and autonomic nervous sys<br>C45 - Malignant neoplasm of retroperitoneum and peritoneum<br>C46 - Malignant neoplasm of other connective and soft tissue<br>C47 - Malignant neoplasm of other connective and soft tissue<br>C47 - Merkel cell carcinoma<br>C48 - Merkel cell carcinoma<br>C49 - Merkel cell carcinoma<br>C50 - Malignant neoplasm of breast<br>C51 - Malignant neoplasm of vulva<br>C52 - Malignant neoplasm of vagina<br>C53 - Malignant neoplasm of cervix uteri<br>C54 - Malignant neoplasm of corpus uteri<br>C55 - Malignant neoplasm of uterus, part unspecified<br>C56 - Malignant neoplasm of ovary<br>C57 - Malignant neoplasm of other and unspecified female genital organs<br>C58 - Malignant neoplasm of placenta<br>C60 - Malignant neoplasm of penis<br>C61 - Malignant neoplasm of prostate<br>C62 - Malignant neoplasm of testis<br>C63 - Malignant neoplasm of other and unspecified male genital organs<br>C64 - Malignant neoplasm of kidney, except renal pelvis<br>C65 - Malignant neoplasm of renal pelvis<br>C66 - Malignant neoplasm of ureter<br>C67 - Malignant neoplasm of bladder<br>C68 - Malignant neoplasm of other and unspecified urinary organs<br>C69 - Malignant neoplasm of eye and adnexa<br>C70 - Malignant neoplasm of meninges<br>C71 - Malignant neoplasm of brain<br>C72 - Malignant neoplasm of spinal cord, cranial nerves and other parts of the central nervous system<br>C73 - Malignant neoplasm of thyroid gland<br>C74 - Malignant neoplasm of adrenal gland<br>C75 - Malignant neoplasm of endo glands and related structures<br>C76 - Secondary malignant neoplasm of other and unspecified sites<br>C76 - Secondary malignant neoplasm of resp and digestive organs
**Valvular disease**	A52 - Late syphilis<br>I05 - Rheumatic mitral valve diseases<br>I06 - Rheumatic aortic valve diseases<br>I07 - Rheumatic tricuspid valve diseases<br>I08 - Multiple valve diseases<br>I09 - Other rheumatic heart diseases<br>I34 - Nonrheumatic mitral valve disorders<br>I35 - Nonrheumatic aortic valve disorders<br>I36 - Nonrheumatic tricuspid valve disorders<br>I37 - Nonrheumatic pulmonary valve disorders<br>I38 - Endocarditis, valve unspecified<br>I39 - Endocarditis and heart valve disorders in diseases classified elsewhere<br>Q23 - Congenital malformations of aortic and mitral valves<br>Z95 - Presence of cardiac and vascular implants and grafts

**Weight loss**	E40 - Kwashiorkor<br>E41 - Nutritional marasmus<br>E42 - Marasmic kwashiorkor<br>E43 - Unspecified severe protein-calorie malnutrition<br>E44 - Protein-calorie malnutrition of moderate and mild degree<br>E45 - Retarded development following protein-calorie malnutrition<br>E46 - Unspecified protein-calorie malnutrition<br>R63 - Symptoms and signs concerning food and fluid intake<br>R64 - Cachexia

Notes:

1. Complicated and uncomplicated diabetes were combined as one condition. Likewise, complicated and uncomplicated hypertension were combined as one condition.
2. ICD-10 descriptions were curated using the *icd* R package.[1]

1. Wasey JO, Frank SM, Rehman MA. icd: Efficient Computation of Comorbidities from ICD Codes Using Sparse Matrix Multiplication in R. Journal of Statistical Software. 2018. Available: https://jackwasey.github.io/icd/articles/efficiency-prebuilt.pdf
