# Supplementary material for: Neurological diagnoses in hospitalized COVID-19 patients associated with adverse outcomes: A multinational cohort study
Source: PLOS Digit Health. 2024 Apr 15;3(4):e0000484. doi: 10.1371/journal.pdig.0000484 (PMC11018281; doi:10.1371/journal.pdig.0000484)
Supplement: S5 Table — Notes: 1. ICD-10 code R42 was listed as peripheral though certain dizziness symptoms could be of central origin. 2. ICD-10 code H54 and ICD-9 code 369 were listed as central but there could be peripheral causes for blindness. (PDF) [file pdig.0000484.s011.pdf]

S5 Table. Major categories of ICD-10 and ICD-9 codes representing central or peripheral nervous system diagnoses in descending order of frequency observed in the adult population.

Nervous System	Neurological Disease Category	ICD Version	Major Category	Description	Number of Adults (% of Adult Cohort)	Number of Children (% of Pediatric Cohort)
Central	Consciousness	ICD-10	R41	Cognitive functions and awareness	7551 (7.3%)	9 (0.4%)
Central	Other	ICD-10	G93	Disorders of the brain	5447 (5.2%)	46 (2.1%)
Central	Seizure	ICD-10	G40	Epilepsy and recurrent seizures	2438 (2.3%)	46 (2.1%)
Peripheral	Dizziness	ICD-10	R42 [1]	Dizziness and giddiness	1225 (1.2%)	4 (0.2%)
Central	Vascular	ICD-10	I67	Cerebrovascular diseases	839 (0.8%)	4 (0.2%)
Peripheral	Neuropathy	ICD-10	R43	Disturbances of smell and taste	830 (0.8%)	3 (0.1%)
Central	Vision	ICD-10	H54 [2]	Blindness and low vision	787 (0.8%)	0 (0%)
Peripheral	Muscle	ICD-10	G72	Myopathies	439 (0.4%)	1 (0%)
Central	Psychiatric	ICD-10	F29	Psychosis	432 (0.4%)	2 (0.1%)
Central	Vascular	ICD-10	G45	Transient cerebral ischemic attacks and related syndromes	407 (0.4%)	0 (0%)
Central	Vascular	ICD-10	I61	Intracerebral hemorrhage (non-traumatic)	323 (0.3%)	0 (0%)
Central	Vascular	ICD-10	I62	Intracranial hemorrhage (non-traumatic)	266 (0.3%)	2 (0.1%)
Central	Coordination	ICD-10	R27	Lack of coordination	235 (0.2%)	1 (0%)
Central	Vascular	ICD-10	G46	Vascular syndromes of brain in cerebrovascular disease	194 (0.2%)	1 (0%)
Central	Headache	ICD-10	G44	Headache syndromes	167 (0.2%)	0 (0%)
Central	Vascular	ICD-10	I60	Subarachnoid hemorrhage (non-traumatic)	126 (0.1%)	1 (0%)
Central	Inflammatory	ICD-10	G04	Encephalitis, myelitis and encephalomyelitis	53 (0.1%)	12 (0.5%)
Peripheral	Neuropathy	ICD-10	G61	Inflammatory polyneuropathy	46 (0%)	0 (0%)
Peripheral	Muscle	ICD-10	M60	Myositis	42 (0%)	1 (0%)
Central	Inflammatory	ICD-10	G03	Meningitis	28 (0%)	4 (0.2%)
Peripheral	Neuropathy	ICD-9	781	Symptoms involving nervous and musculoskeletal systems	28 (0%)	0 (0%)
Peripheral	Neuropathy	ICD-9	357	Inflammatory and toxic neuropathy	24 (0%)	0 (0%)
Central	Other	ICD-9	780	Symptoms (general)	23 (0%)	0 (0%)
Central	Consciousness	ICD-9	799	Ill-defined and unknown causes of morbidity and mortality	15 (0%)	0 (0%)
Central	Vascular	ICD-9	431	Intracerebral hemorrhage (non-traumatic)	8 (0%)	0 (0%)
Central	Seizure	ICD-9	345	Epilepsy and recurrent seizures	7 (0%)	0 (0%)
Central	Other	ICD-9	348	Conditions of brain	7 (0%)	0 (0%)
Central	Psychiatric	ICD-9	298	Psychoses	6 (0%)	0 (0%)
Central	Other	ICD-9	331	Cerebral degenerations	6 (0%)	0 (0%)
Central	Vascular	ICD-9	435	Transient cerebral ischemia	6 (0%)	0 (0%)
Peripheral	Muscle	ICD-9	728	Disorders of muscle, ligament, and fascia	2 (0%)	0 (0%)
Central	Vascular	ICD-9	437	Cerebrovascular disease (other, but ill-defined)	1 (0%)	0 (0%)
Central	Vascular	ICD-9	430	Subarachnoid hemorrhage (non-traumatic)	0 (0%)	0 (0%)

Central	Vascular	ICD-9	432	Intracranial hemorrhage (non-traumatic)	0 (0%)	0 (0%)
Peripheral	Muscle	ICD-9	729	Disorders of soft tissue	0 (0%)	0 (0%)
Central	Headache	ICD-9	784	Symptoms involving head and neck	0 (0%)	0 (0%)
Central	Consciousness	ICD-9	797	Senility without mention of psychosis	0 (0%)	0 (0%)
Central	Inflammatory	ICD-9	323	Encephalitis myelitis and encephalomyelitis	0 (0%)	0 (0%)
Central	Vascular	ICD-9	436	Cerebrovascular disease (acute, but ill-defined)	0 (0%)	0 (0%)
Peripheral	Neuropathy	ICD-10	G65	Inflammatory and toxic polyneuropathies sequelae	0 (0%)	0 (0%)
Peripheral	Muscle	ICD-9	40	Bacterial diseases	0 (0%)	0 (0%)
Central	Headache	ICD-9	307	Symptoms or syndromes (not elsewhere classified)	0 (0%)	0 (0%)
Central	Inflammatory	ICD-9	320	Meningitis (bacterial)	0 (0%)	0 (0%)
Central	Inflammatory	ICD-9	321	Meningitis (nonbacterial)	0 (0%)	0 (0%)
Central	Inflammatory	ICD-9	322	Meningitis (unspecified)	0 (0%)	0 (0%)
Central	Other	ICD-9	330	Cerebral degenerations (pediatric)	0 (0%)	0 (0%)
Central	Headache	ICD-9	339	Headache syndromes	0 (0%)	0 (0%)
Peripheral	Muscle	ICD-9	359	Muscular dystrophies and other myopathies	0 (0%)	0 (0%)
Central	Vision	ICD-9	369[2]	Blindness and low vision	0 (0%)	0 (0%)
Peripheral	Vision/smell/taste	ICD-9	V41	Problems with special senses and other special functions	0 (0%)	0 (0%)

Notes:

1. ICD-10 code R42 was listed as peripheral though certain dizziness symptoms could be of central origin.
2. ICD-10 code H54 and ICD-9 code 369 were listed as central but there could be peripheral causes for blindness.
