# Supplementary material for: Neurological diagnoses in hospitalized COVID-19 patients associated with adverse outcomes: A multinational cohort study
Source: PLOS Digit Health. 2024 Apr 15;3(4):e0000484. doi: 10.1371/journal.pdig.0000484 (PMC11018281; doi:10.1371/journal.pdig.0000484)
Supplement: S6 Table — Notes: 1. We could not conduct similar analyses in children due to the low frequency of pre-admission health conditions in pediatric patients. Refer to S7 Table for the overall count and percentage of adult and pediatric patients with pre-admission health conditions as stratified by neurological status. 2. Refer to S3–S4 Tables for detailed descriptions of ICD codes comprising each component of the Elixhauser Comorbidity Index. 3. Complicated and uncomplicated diabetes were combined as one condition. Likewise, complicated and uncomplicated hypertension were combined as one condition. (PDF) [file pdig.0000484.s012.pdf]

S6 Table. Relative risk (RR) of a concurrent central nervous system (CNS) or peripheral nervous system (PNS) diagnosis during acute COVID-19 hospitalizations with respect to each pre-admission health condition in descending order of the RR for the CNS diagnosis in adults.[1]

**Pre-Admission Health Condition [2]**	**RR CNS (95% CI)**	**RR PNS (95% CI)**
Neurological disorders	3.23 (3.13 , 3.33)	0.63 (0.53 , 0.74)
Paralysis	2.55 (2.38 , 2.74)	0.47 (0.3 , 0.75)
Psychoses	2.25 (2.13 , 2.37)	0.27 (0.18 , 0.4)
Fluid and electrolyte disorders	1.87 (1.8 , 1.94)	0.99 (0.87 , 1.11)
Hypertension[3]	1.83 (1.78 , 1.88)	1.06 (0.99 , 1.15)
Weight loss	1.83 (1.74 , 1.91)	0.67 (0.55 , 0.82)
Alcohol abuse	1.74 (1.69 , 1.8)	1.13 (1.05 , 1.23)
Cardiac arrhythmias	1.73 (1.68 , 1.79)	1.09 (0.99 , 1.19)
Drug abuse	1.72 (1.67 , 1.77)	1.15 (1.06 , 1.25)
Peripheral vascular disorders	1.71 (1.65 , 1.78)	1 (0.9 , 1.13)
Depression	1.69 (1.63 , 1.74)	1.25 (1.14 , 1.36)
Congestive heart failure	1.68 (1.63 , 1.74)	0.99 (0.89 , 1.09)
Renal failure	1.66 (1.6 , 1.72)	1.01 (0.91 , 1.12)
Coagulopathy	1.64 (1.53 , 1.75)	0.71 (0.55 , 0.91)
Deficiency anemias	1.61 (1.53 , 1.69)	1.07 (0.92 , 1.24)
Valvular disease	1.59 (1.53 , 1.65)	1.02 (0.9 , 1.15)
Diabetes[3]	1.58 (1.53 , 1.63)	1.11 (1.02 , 1.21)
Hypothyroidism	1.55 (1.47 , 1.63)	1.04 (0.9 , 1.21)
Blood loss anemia	1.53 (1.45 , 1.62)	0.97 (0.81 , 1.15)
Peptic ulcer disease excluding bleeding	1.39 (1.21 , 1.6)	0.46 (0.25 , 0.86)
Chronic pulmonary disease	1.36 (1.31 , 1.41)	0.99 (0.89 , 1.1)
Metastatic cancer	1.36 (1.23 , 1.5)	0.76 (0.55 , 1.05)
Solid tumor without metastasis	1.35 (1.28 , 1.42)	0.99 (0.85 , 1.14)
Pulmonary circulation disorders	1.34 (1.24 , 1.44)	0.76 (0.6 , 0.97)
Liver disease	1.21 (1.14 , 1.27)	1.17 (1.02 , 1.34)
Rheumatoid arthritis/collagen vascular diseases	1.18 (1.09 , 1.27)	1.07 (0.87 , 1.32)
Lymphoma	1.08 (0.95 , 1.23)	0.73 (0.49 , 1.08)
Obesity	1.04 (0.99 , 1.09)	1.18 (1.06 , 1.32)
HIV/AIDS	0.96 (0.76 , 1.21)	0.08 (0.01 , 0.6)

Notes:

1. We could not conduct similar analyses in children due to the low frequency of pre-admission health conditions in pediatric patients. Refer to S7 Table for the overall count and percentage of adult and pediatric patients with pre-admission health conditions as stratified by neurological status.
2. Refer to S3-S4 Tables for detailed descriptions of ICD codes comprising each component of the Elixhauser Comorbidity Index.
3. Complicated and uncomplicated diabetes were combined as one condition. Likewise, complicated and uncomplicated hypertension were combined as one condition.
