# Supplementary material for: Neurological diagnoses in hospitalized COVID-19 patients associated with adverse outcomes: A multinational cohort study
Source: PLOS Digit Health. 2024 Apr 15;3(4):e0000484. doi: 10.1371/journal.pdig.0000484 (PMC11018281; doi:10.1371/journal.pdig.0000484)
Supplement: S7 Table — Notes: 1. Neurological status during acute COVID-19 hospitalization: central nervous system diagnosis (CNS), peripheral nervous system diagnosis (PNS), no neurological condition (NNC). 2. Refer to S3–S4 Tables for detailed descriptions of ICD codes comprising each component of the Elixhauser Comorbidity Index. 3. N = the total number of adult or pediatric patients with the pre-admission health condition; the corresponding percentage is out of the total adult or pediatric population. 4. Percentages in the NNC, CNS, and PNS columns reflect the percent of patients with the respective neurological status who have the indicated pre-admission health condition. 5. Complicated and uncomplicated diabetes were combined as one condition. Likewise, complicated and uncomplicated hypertension were combined as one condition. (PDF) [file pdig.0000484.s013.pdf]

S7 Table. Count and percentage of adult and pediatric patients with pre-admission health conditions as stratified by concurrent neurological status during acute COVID-19 hospitalization [1].

Pre-Admission Health Condition [2]	Population	Total, N (%) [3]	NNC, N (%) [4]	CNS, N (%) [4]	PNS, N (%) [4]
**Alcohol abuse**	Adult	29918 (29%)	22879 (26.5 %)	6169 (41.3 %)	870 (31.4 %)
	Pediatric	212 (10%)	186 (9.2 %)	25 (15.3 %)	1 (6.2 %)
**Blood loss anemia**	Adult	4883 (5%)	3710 (4.3 %)	1047 (7 %)	126 (4.5 %)
	Pediatric	23 (1%)	20 (1 %)	2 (1.2 %)	1 (6.2 %)
**Cardiac arrhythmias**	Adult	19196 (18%)	14441 (16.7 %)	4207 (28.2 %)	548 (19.8 %)
	Pediatric	109 (5%)	94 (4.7 %)	12 (7.4 %)	3 (18.8 %)
**Chronic pulmonary disease**	Adult	14119 (14%)	11121 (12.9 %)	2626 (17.6 %)	372 (13.4 %)
	Pediatric	71 (3%)	62 (3.1 %)	9 (5.5 %)	0 (0 %)
**Coagulopathy**	Adult	3155 (3%)	2367 (2.7 %)	728 (4.9 %)	60 (2.2 %)
	Pediatric	41 (2%)	36 (1.8 %)	4 (2.5 %)	1 (6.2 %)
**Congestive heart failure**	Adult	17895 (17%)	13555 (15.7 %)	3869 (25.9 %)	471 (17 %)
	Pediatric	17 (1%)	16 (0.8 %)	1 (0.6 %)	0 (0 %)
**Deficiency anemias**	Adult	6143 (6%)	4601 (5.3 %)	1368 (9.2 %)	174 (6.3 %)
	Pediatric	23 (1%)	20 (1 %)	2 (1.2 %)	1 (6.2 %)
**Depression**	Adult	18794 (18%)	14149 (16.4 %)	4047 (27.1 %)	598 (21.6 %)
	Pediatric	78 (4%)	72 (3.6 %)	5 (3.1 %)	1 (6.2 %)
**Diabetes [5]**	Adult	21952 (21%)	16888 (19.6 %)	4429 (29.6 %)	635 (22.9 %)
	Pediatric	17 (1%)	17 (0.8 %)	0 (0 %)	0 (0 %)
**Drug abuse**	Adult	26389 (25%)	20097 (23.3 %)	5512 (36.9 %)	780 (28.1 %)
	Pediatric	207 (9%)	190 (9.4 %)	16 (9.8 %)	1 (6.2 %)
**Fluid and electrolyte disorders**	Adult	10565 (10%)	7680 (8.9 %)	2607 (17.5 %)	278 (10 %)
	Pediatric	74 (3%)	69 (3.4 %)	4 (2.5 %)	1 (6.2 %)
**HIV/AIDS**	Adult	444 (0%)	382 (0.4 %)	61 (0.4 %)	1 (0 %)
	Pediatric	0 (0%)	0 (0 %)	0 (0 %)	0 (0 %)
**Hypertension [5]**	Adult	35566 (34%)	27302 (31.6 %)	7277 (48.7 %)	987 (35.6 %)
	Pediatric	21 (1%)	21 (1 %)	0 (0 %)	0 (0 %)
**Hypothyroidism**	Adult	6419 (6%)	4860 (5.6 %)	1381 (9.2 %)	178 (6.4 %)
	Pediatric	8 (0%)	7 (0.3 %)	1 (0.6 %)	0 (0 %)
**Liver disease**	Adult	6683 (6%)	5335 (6.2 %)	1142 (7.6 %)	206 (7.4 %)
	Pediatric	34 (2%)	32 (1.6 %)	2 (1.2 %)	0 (0 %)
**Lymphoma**	Adult	1283 (1%)	1059 (1.2 %)	199 (1.3 %)	25 (0.9 %)
	Pediatric	7 (0%)	7 (0.3 %)	0 (0 %)	0 (0 %)
**Metastatic cancer**	Adult	1773 (2%)	1393 (1.6 %)	344 (2.3 %)	36 (1.3 %)
	Pediatric	6 (0%)	5 (0.2 %)	0 (0 %)	1 (6.2 %)
**Neurological disorders**	Adult	8888 (9%)	5274 (6.1 %)	3461 (23.2 %)	153 (5.5 %)
	Pediatric	87 (4%)	46 (2.3 %)	41 (25.2 %)	0 (0 %)
**Obesity**	Adult	11536 (11%)	9471 (11 %)	1709 (11.4 %)	356 (12.8 %)
	Pediatric	28 (1%)	26 (1.3 %)	2 (1.2 %)	0 (0 %)
	Adult	1424 (1%)	895 (1 %)	511 (3.4 %)	18 (0.6 %)

**Paralysis**	Pediatric	30 (1%)	12 (0.6 %)	18 (11 %)	0 (0 %)
**Peptic ulcer disease excluding bleeding**	Adult	804 (1%)	634 (0.7 %)	160 (1.1 %)	10 (0.4 %)
	Pediatric	1 (0%)	1 (0 %)	0 (0 %)	0 (0 %)
**Peripheral vascular disorders**	Adult	12075 (12%)	9011 (10.4 %)	2741 (18.3 %)	323 (11.7 %)
	Pediatric	46 (2%)	39 (1.9 %)	6 (3.7 %)	1 (6.2 %)
**Psychoses**	Adult	3258 (3%)	2223 (2.6 %)	1011 (6.8 %)	24 (0.9 %)
	Pediatric	11 (1%)	10 (0.5 %)	1 (0.6 %)	0 (0 %)
**Pulmonary circulation disorders**	Adult	3218 (3%)	2541 (2.9 %)	611 (4.1 %)	66 (2.4 %)
	Pediatric	8 (0%)	7 (0.3 %)	1 (0.6 %)	0 (0 %)
**Renal failure**	Adult	15239 (15%)	11521 (13.3 %)	3310 (22.2 %)	408 (14.7 %)
	Pediatric	65 (3%)	53 (2.6 %)	11 (6.7 %)	1 (6.2 %)
**Rheumatoid arthritis / collagen vascular diseases**	Adult	3097 (3%)	2489 (2.9 %)	520 (3.5 %)	88 (3.2 %)
	Pediatric	5 (0%)	3 (0.1 %)	1 (0.6 %)	1 (6.2 %)
**Solid tumor without metastasis**	Adult	7061 (7%)	5537 (6.4 %)	1338 (9 %)	186 (6.7 %)
	Pediatric	21 (1%)	20 (1 %)	0 (0 %)	1 (6.2 %)
**Valvular disease**	Adult	10089 (10%)	7639 (8.8 %)	2177 (14.6 %)	273 (9.8 %)
	Pediatric	53 (2%)	45 (2.2 %)	7 (4.3 %)	1 (6.2 %)
**Weight loss**	Adult	5703 (5%)	4169 (4.8 %)	1430 (9.6 %)	104 (3.8 %)
	Pediatric	123 (6%)	109 (5.4 %)	13 (8 %)	1 (6.2 %)

Notes:

1. Neurological status during acute COVID-19 hospitalization: central nervous system diagnosis (CNS), peripheral nervous system diagnosis (PNS), no neurological condition (NNC).
2. Refer to S3-S4 Tables for detailed descriptions of ICD codes comprising each component of the Elixhauser Comorbidity Index.
3. N=the total number of adult or pediatric patients with the pre-admission health condition; the corresponding percentage is out of the total adult or pediatric population.
4. Percentages in the NNC, CNS, and PNS columns reflect the percent of patients with the respective neurological status who have the indicated pre-admission health condition.
5. Complicated and uncomplicated diabetes were combined as one condition. Likewise, complicated and uncomplicated hypertension were combined as one condition.
