# Supplementary material for: Neurological diagnoses in hospitalized COVID-19 patients associated with adverse outcomes: A multinational cohort study
Source: PLOS Digit Health. 2024 Apr 15;3(4):e0000484. doi: 10.1371/journal.pdig.0000484 (PMC11018281; doi:10.1371/journal.pdig.0000484)
Supplement: S8 Table — Notes: 1. We report each healthcare system’s total number of severe and neurological patients used to calculate the PMI at each healthcare system. PMI >0 indicates more frequent co-occurrence (between a CNS or a PNS diagnosis and severe COVID-19 status) than independent assumptions. 2. Severe COVID-19 status was based on previously published computable phenotypes, including diagnosis of pneumonia and/or acute respiratory distress syndrome, need for mechanical ventilation, sedation, and/or medication administration for shock [1]. 3. 95% confidence intervals were estimated using 500 bootstrapped samples. 4. Bold findings indicate statistically significant results. Supplemental Citation 1. Klann, J. G. et al. Validation of an internationally derived patient severity phenotype to support COVID-19 analytics from electronic health record data. Journal of the American Medical Informatics Association 28: 1411–1420 (2021). (PDF) [file pdig.0000484.s014.pdf]

S8 Table. Pointwise mutual information (PMI) of a central nervous system (CNS) or peripheral nervous system (PNS) diagnosis co-occurring with severe COVID-19 disease during acute COVID-19 hospitalization.

**Healthcare System [1]**	**N (%) Severe [2]**	**N (%) CNS & Severe**	**N (%) PNS & Severe**	**N (%) NNC & Severe**	**CNS PMI (95% CI) [3, 4]**	**PNS PMI (95% CI) [3, 4]**
APHP	1964 (8%)	232 (10.9%)	70 (15.8%)	1662 (7.6%)	**0.45 (0.28, 0.61)**	0.39 (-0.28, 1.06)
FRBDX	440 (39.9%)	140 (47.5%)	40 (37%)	260 (37.2%)	**0.41 (0.07, 0.75)**	-0.17 (-8.99, 8.64)
H12O	428 (11.7%)	37 (22%)	0 (0%)	391 (11.2%)	0.67 (-7.68, 9.02)	**7.99 (4.97, 11)**
HPG23	524 (20.7%)	27 (27.8%)	21 (51.2%)	476 (19.9%)	7.36 (-14.7, 29.43)	13.12 (-7.36, 33.61)
MGB	1185 (22.9%)	296 (29.7%)	67 (19.3%)	822 (21.5%)	**0.42 (0.13, 0.72)**	-0.11 (-0.42, 0.21)
NWU	1350 (24.5%)	325 (46.3%)	46 (27.7%)	979 (21.1%)	**0.78 (0.65, 0.91)**	0.18 (-0.17, 0.53)
UCLA	359 (23.3%)	92 (30.7%)	11 (21.6%)	256 (21.5%)	**5.94 (2.85, 9.03)**	4.63 (-2.96, 12.22)
UKY	246 (35%)	73 (50.7%)	7 (38.9%)	166 (30.7%)	**7.01 (0.05, 13.97)**	**8.24 (1.92, 14.56)**
UMICH	263 (18.2%)	32 (37.6%)	7 (19.4%)	224 (16.9%)	0.73 (-6.87, 8.32)	-0.31 (-3.91, 3.29)
UPENN	1247 (17%)	79 (28.9%)	13 (18.8%)	1155 (16.6%)	2.62 (-0.76, 6)	**3.39 (0.62, 6.15)**
UPITT	2010 (23.1%)	340 (30.9%)	25 (20.3%)	1645 (22%)	2.12 (-1.2, 5.45)	0.29 (-1.31, 1.89)
VA1	868 (22.7%)	258 (34%)	24 (20.3%)	586 (19.9%)	**0.48 (0.18, 0.79)**	0.2 (-7.9, 8.3)
VA2	1086 (23.8%)	336 (34%)	38 (24.8%)	712 (20.8%)	**0.48 (0.37, 0.59)**	-0.06 (-1.28, 1.17)
VA3	1065 (20.4%)	289 (29.3%)	19 (14%)	757 (18.5%)	**0.38 (0.13, 0.63)**	-0.51 (-1.08, 0.06)
VA4	980 (27%)	261 (34%)	21 (23.6%)	698 (25.2%)	**0.33 (0.14, 0.51)**	-0.01 (-1.38, 1.35)
VA5	865 (24%)	245 (34.9%)	23 (22.8%)	597 (21.4%)	**0.46 (0.32, 0.6)**	**3.97 (0.31, 7.62)**

Notes:

1. We report each healthcare system's total number of severe and neurological patients used to calculate the PMI at each healthcare system. PMI >0 indicates more frequent co-occurrence (between a CNS or a PNS diagnosis and severe COVID-19 status) than independent assumptions.
2. Severe COVID-19 status was based on previously published computable phenotypes, including diagnosis of pneumonia and/or acute respiratory distress syndrome, need for mechanical ventilation, sedation, and/or medication administration for shock.[1]
3. 95% confidence intervals were estimated using 500 bootstrapped samples.
4. Bold findings indicate statistically significant results.

1. Klann JG, Estiri H, Weber GM, Moal B, Avillach P, Hong C, et al. Validation of an internationally derived patient severity phenotype to support COVID-19 analytics from electronic health record data. Journal of the American Medical Informatics Association. 2021;28: 1411–1420. doi:10.1093/jamia/ocab018
