# Supplementary material for: Neurological diagnoses in hospitalized COVID-19 patients associated with adverse outcomes: A multinational cohort study
Source: PLOS Digit Health. 2024 Apr 15;3(4):e0000484. doi: 10.1371/journal.pdig.0000484 (PMC11018281; doi:10.1371/journal.pdig.0000484)
Supplement: S9 Table — Notes: 1. Neurological status during acute COVID-19 hospitalization: NNC = No Neurological Condition; CNS = Central Nervous System diagnosis; PNS = Peripheral Nervous System diagnosis. 2. P-values were adjusted with the Benjamini-Hochberg method to control the false discovery rate when evaluating the distribution (categorical variables) or means (continuous variables) of characteristics stratified by neurological status. P-values < .05 were deemed significant and designated with asterisk (*). 3. Continuous variables reflect the overall cohort mean and standard deviation (SD) of the median values reported by each healthcare system. 4. We report the top 4 most frequent pre-admission health conditions (i.e., comorbidities) in the adult population. Please see S7 Table for all pre-admission health conditions stratified by neurological status during acute COVID-19 hospitalization. 5. We could not compute chi-square statistic for the mean number of pre-admission CNS codes, due to every healthcare system having a median value of 0. (PDF) [file pdig.0000484.s015.pdf]

S9 Table. Adult Population Characteristics

	Entire Cohort	NNC [1]	CNS [1]	PNS [1]	P-value [2]
**All Patients, N (% of cohort)**	104031 (100%)	86321 (83%)	14938 (14.4%)	2772 (2.7%)	
**Sex, N (%)**					<.001*
Female	36564	31445 (36.4%)	4185 (28%)	934 (33.7%)	
Male	67466	54875 (63.6%)	10753 (72%)	1838 (66.3%)	
Unknown Sex	1	1 (0%)	0 (0%)	0 (0%)	
**Age, N (%)**					
18-25	2328	2138 (2.5%)	145 (1%)	45 (1.6%)	<.001*
26-49	17799	16122 (18.7%)	1166 (7.8%)	511 (18.5%)	<.001*
50-69	36885	31556 (36.6%)	4169 (28%)	1160 (42.1%)	<.001*
70-79	24860	19672 (22.8%)	4508 (30.2%)	680 (24.7%)	<.001*
80+	22094	16813 (19.5%)	4920 (33%)	361 (13.1%)	<.001*
**Race, N (%)**					
American Indian	349	294 (0.3%)	55 (0.4%)	0 (0%)	.01*
Asian	1633	1412 (1.6%)	196 (1.3%)	25 (0.9%)	<.001*
Black	16631	13202 (15.3%)	2979 (20%)	450 (16.5%)	<.001*
Hawaiian/Pacific Islander	296	254 (0.3%)	41 (0.3%)	1 (0%)	.051
Hispanic/Latino	850	799 (0.9%)	29 (0.2%)	22 (0.8%)	<.001*
White	41206	32752 (38%)	7354 (49.3%)	1100 (40.3%)	<.001*
Other / Not Recorded	42971	37587 (43.6%)	4254 (28.5%)	1130 (41.4%)	<.001*
**Pre-admission Elixhauser score, Mean (SD) [3]**	0.3 (0.7)	0.2 (0.5)	1.4 (2)	0.6 (1.7)	.074
**Pre-admission Conditions (Selected), N (%) [4]**					
Hypertension	35566	27302 (31.6 %)	7277 (48.7 %)	987 (35.6 %)	<.001*
Alcohol abuse	29918	22879 (26.5 %)	6169 (41.3 %)	870 (31.4 %)	<.001*
Drug abuse	26389	20097 (23.3 %)	5512 (36.9 %)	780 (28.1 %)	<.001*
Diabetes	21952	16888 (19.6 %)	4429 (29.6 %)	635 (22.9 %)	<.001*
**Number of pre-admission CNS codes, Mean (SD) [3]**	0 (0)	0 (0)	0 (0)	0 (0)	NA[5]
**Number of pre-admission PNS codes, Mean (SD) [3]**	0 (0)	0 (0)	0 (0)	0 (0.1)	.397
**Severe Status, N (%)**					<.001*
Non-Severe	64578	56181 (65.1%)	6935 (46.4%)	1462 (52.6%)	
Severe	39468	30140 (34.9%)	8008 (53.6%)	1320 (47.4%)	
**Time to severe status, Mean days (SD) [3]**	0.6 (1)	0.8 (1.3)	0.4 (0.7)	0.8 (1.1)	.487
**Survival, N (%)**					<.001*
Alive	85212	72388 (83.9%)	10247 (68.6%)	2577 (93.1%)	
Deceased	18820	13933 (16.1%)	4696 (31.4%)	191 (6.9%)	
**Time to death, Mean days (SD) [3]**	15.3 (6.4)	15.9 (5.8)	22.4 (32.5)	36.9 (41.4)	.024*
**Time to discharge, Mean days (SD) [3]**	8.4 (4.2)	6.5 (3.6)	12.8 (6.1)	14.5 (18.3)	<.001*
**Readmission, N (%)**					<.001*
Not Readmitted	89802	75170 (87.1%)	12312 (82.4%)	2320 (83.5%)	
Readmitted	14237	11151 (12.9%)	2626 (17.6%)	460 (16.5%)	

**Time to first readmission, Mean days (SD) [3]**	28.1 (20.3)	26.4 (17.6)	31.9 (33.7)	38.1 (28.4)	.495
**Number of readmissions, Mean (SD) [3]**	0.1 (0.3)	0 (0)	0.1 (0.3)	0.1 (0.3)	.391

Notes:

1. Neurological status during acute COVID-19 hospitalization: NNC=No Neurological Condition; CNS=Central Nervous System diagnosis; PNS=Peripheral Nervous System diagnosis.
2. P-values were adjusted with the Benjamini-Hochberg method to control the false discovery rate when evaluating the distribution (categorical variables) or means (continuous variables) of characteristics stratified by neurological status. P-values < .05 were deemed significant and designated with asterisk (*).
3. Continuous variables reflect the overall cohort mean and standard deviation (SD) of the median values reported by each healthcare system.
4. We report the top 4 most frequent pre-admission health conditions (*i.e.*, comorbidities) in the adult population. Please see S7 Table for all pre-admission health conditions stratified by neurological status during acute COVID-19 hospitalization.
5. We could not compute chi-square statistic for the mean number of pre-admission CNS codes, due to every healthcare system having a median value of 0.
