# Supplementary material for: Neurological diagnoses in hospitalized COVID-19 patients associated with adverse outcomes: A multinational cohort study
Source: PLOS Digit Health. 2024 Apr 15;3(4):e0000484. doi: 10.1371/journal.pdig.0000484 (PMC11018281; doi:10.1371/journal.pdig.0000484)
Supplement: S10 Table — Notes: 1. Neurological status during acute COVID-19 hospitalization: NNC = No Neurological Condition; CNS = Central Nervous System diagnosis; PNS = Peripheral Nervous System diagnosis. 2. P-values were adjusted with the Benjamini-Hochberg method to control the false discovery rate when evaluating the distribution (categorical variables) or means (continuous variables) of characteristics stratified by neurological status. P-values < .05 were deemed significant and designated with asterisk (*). 3. Continuous variables reflect the overall cohort mean and standard deviation (SD) of the median values reported by each healthcare system. 4. We report the top 4 most frequent pre-admission health conditions (i.e., comorbidities) in the pediatric population. Please see S7 Table for all pre-admission health conditions stratified by neurological status during acute COVID-19 hospitalization. (PDF) [file pdig.0000484.s016.pdf]

S10 Table. Pediatric Population Characteristics

	Entire Cohort	NNC [1]	CNS [1]	PNS [1]	P-value [2]
**All Patients, N (% of cohort)**	2198 (100%)	2019 (91.9%)	163 (7.4%)	16 (0.73%)	
**Sex, N (%)**					.074
Female	1026	954 (47.3%)	62 (38%)	10 (62.5%)	
Male	1172	1065 (52.7%)	101 (62%)	6 (37.5%)	
**Age, N (%)**					
0-2	769	747 (37.3%)	22 (15.8%)	0 (0%)	<.001*
3-5	275	251 (12.5%)	24 (17.3%)	0 (0%)	.283
6-11	403	368 (18.4%)	33 (23.7%)	2 (16.7%)	.763
12-17	707	637 (31.8%)	60 (43.2%)	10 (83.3%)	.039*
**Race, N (%)**					
American Indian	1	1 (0%)	0 (0%)	0 (0%)	.957
Asian	61	52 (2.6%)	9 (5.8%)	0 (0%)	.119
Black	184	166 (8.3%)	16 (10.4%)	2 (12.5%)	.763
Hawaiian/Pacific Islander	1	1 (0%)	0 (0%)	0 (0%)	.957
Hispanic/Latino	20	20 (1%)	0 (0%)	0 (0%)	.502
White	665	608 (30.2%)	47 (30.5%)	10 (62.5%)	.048*
Other / Not Recorded	1249	1163 (57.8%)	82 (53.2%)	4 (25%)	.024*
**Pre-admission Elixhauser score, Mean (SD) [3]**	2.2 (4.5)	0 (0)	4.4 (9.2)	2.5 (3.6)	.074
**Pre-admission Conditions (Selected), N (%) [4]**					
Alcohol abuse	212	186 (9.2 %)	25 (15.3 %)	1 (6.2 %)	<.001*
Drug abuse	207	190 (9.4 %)	16 (9.8 %)	1 (6.2 %)	<.001*
Weight loss	123	109 (5.4 %)	13 (8 %)	1 (6.2 %)	<.001*
Cardiac arrhythmias	109	94 (4.7 %)	12 (7.4 %)	3 (18.8 %)	<.001*
**Number of pre-admission CNS codes, Mean (SD) [3]**	0.2 (0.7)	0.1 (0.2)	1.9 (3.9)	0 (0)	.211
**Number of pre-admission PNS codes, Mean (SD) [3]**	0 (0)	0 (0)	0 (0)	0.2 (0.6)	.067
**Severe Status, N (%)**					<.001*
Non-Severe	1674	1590 (78.5%)	72 (48.6%)	12 (70.6%)	
Severe	517	436 (21.5%)	76 (51.4%)	5 (29.4%)	
**Time to severe status, Mean days (SD) [3]**	2.4 (5.3)	3.8 (9.5)	10.8 (33.1)	0.9 (2.5)	.269
**Survival, N (%)**					<.001*
Alive	2164	2001 (99%)	145 (94.2%)	18 (100%)	
Deceased	29	20 (1%)	9 (5.8%)	0 (0%)	
**Time to death, Mean days (SD) [3]**	12.6 (36.3)	53.5 (76.3)	5.8 (10.2)	95 (268.7)	.269
**Time to discharge, Mean days (SD) [3]**	5.1 (4.2)	3.8 (4.2)	5.8 (2.7)	5.9 (4.5)	.082
**Readmission, N (%)**					.957
Not Readmitted	1844	1707 (84.8%)	123 (83.7%)	14 (82.4%)	
Readmitted	332	305 (15.2%)	24 (16.3%)	3 (17.6%)	
**Time to first readmission, Mean days (SD) [3]**	20 (20.2)	20.1 (19)	75.3 (86)	14.4 (22.8)	.171
**Number of readmissions, Mean (SD) [3]**	0.1 (0.3)	0 (0)	0.2 (0.4)	0.4 (0.5)	.082

Notes:

1. Neurological status during acute COVID-19 hospitalization: NNC=No Neurological Condition; CNS=Central Nervous System diagnosis; PNS=Peripheral Nervous System diagnosis.
2. P-values were adjusted with the Benjamini-Hochberg method to control the false discovery rate when evaluating the distribution (categorical variables) or means (continuous variables) of characteristics stratified by neurological status. P-values < .05 were deemed significant and designated with asterisk (*).
3. Continuous variables reflect the overall cohort mean and standard deviation (SD) of the median values reported by each healthcare system.
4. We report the top 4 most frequent pre-admission health conditions (*i.e.*, comorbidities) in the pediatric population. Please see S7 Table for all pre-admission health conditions stratified by neurological status during acute COVID-19 hospitalization.
