# Supplementary material for: Neurological diagnoses in hospitalized COVID-19 patients associated with adverse outcomes: A multinational cohort study
Source: PLOS Digit Health. 2024 Apr 15;3(4):e0000484. doi: 10.1371/journal.pdig.0000484 (PMC11018281; doi:10.1371/journal.pdig.0000484)
Supplement: S1 Methods — (PDF) [file pdig.0000484.s017.pdf]

S1 Methods. Protecting Patient Confidentiality

To preserve healthcare system-specific confidentiality and to reduce the risk of patient re-identification across different systems, several of the 4CE consortium participating healthcare systems obfuscated small counts based on the local institutional approval as previously described.[1] Obfuscation occurred by masking numbers below a predefined threshold and through blurring. For example, if a system had a mask threshold of ten, all counts below ten were converted to zero. Additionally, if a participating healthcare system had a blurring range of three, a random number from -3 to 3 was generated and all counts were adjusted up or down by that number. Importantly, the Cox-proportional hazards models and covariate-adjusted survival curves were performed on the original source data of each healthcare system *without* obfuscation. In the meta-analysis (Fig 6 in the main text), the estimated number of patients at risk (at 0, 30, 60, and 90 days) for clinical events would be insignificantly affected by obfuscation. Given the large size of the overall study population, obfuscation at some of the participating healthcare system would have minimal, if any, impact on the total patient counts used to evaluate baseline and clinical characteristics or pre-existing health conditions among neurological strata or the survival analyses.

1. Brat GA, Weber GM, Gehlenborg N, Avillach P, Palmer NP, Chiovato L, et al. International electronic health record-derived COVID-19 clinical course profiles: the 4CE consortium. npj Digital Medicine. 2020;3. doi:10.1038/s41746-020-00308-0
