# Supplementary material for: Neurological diagnoses in hospitalized COVID-19 patients associated with adverse outcomes: A multinational cohort study
Source: PLOS Digit Health. 2024 Apr 15;3(4):e0000484. doi: 10.1371/journal.pdig.0000484 (PMC11018281; doi:10.1371/journal.pdig.0000484)
Supplement: S2 Methods — (PDF) [file pdig.0000484.s018.pdf]

S2 Methods. Logistic Principal Component Analysis.

As a sensitivity analysis, we evaluated previously published method of using Logistic Principal Component Analysis to adjust for a patient's comorbidity burden.[1] At each participating 4CE healthcare system, we computed the top 10 principal components using logistic principal component analysis (LPCA). LPCA was performed on the matrix of n patients x 29 Elixhauser Comorbidity Index (ECI) comorbidities.

Assessment using data from the participating healthcare systems found that the top 10 PCA components explained > 70% of the expected model deviance, consistent with prior publication of this method. In supplementary analysis (S4-S5 Figures), we observed that using the LPCA approach had comparable model estimates as survival models that controlled for comorbidity burden using either the individual covariates of the ECI or the weighted summary score.

1. Renson A, Bjurlin MA. The Charlson Index Is Insufficient to Control for Comorbidities in a National Trauma Registry. Journal of Surgical Research. 2019;236: 319–325. doi:10.1016/j.jss.2018.07.072
