# Supplementary material for: Correction: Neurological diagnoses in hospitalized COVID-19 patients associated with adverse outcomes: A multinational cohort study
Source: PLOS Digit Health. 2025 Jul 22;4(7):e0000957. doi: 10.1371/journal.pdig.0000957 (PMC12282889; doi:10.1371/journal.pdig.0000957)
Supplement: S1 Methods — (PDF) [file pdig.0000957.s001.pdf]

S1 Methods. Protecting Patient Confidentiality

To preserve healthcare system-specific confidentiality and to reduce the risk of patient re-identification across different systems, several of the 4CE consortium participating healthcare systems obfuscated small counts based on the local institutional approval as previously described.[1] Obfuscation occurred by masking numbers below a predefined threshold and / or through blurring. For example, if a system had a mask threshold of ten, all counts below ten were converted to zero. As another example, if a participating healthcare system had a blurring range of three, a random number from -3 to 3 was generated and all counts were adjusted up or down by that number. The **main Figure 2** displays the counts of patient demographics and clinical characteristics at each healthcare system *after* obfuscation. As such, the overall counts and the counts of patients stratified by demographic factors may slightly vary from the true number. This difference is more noticeable in healthcare systems with a smaller sample size (*e.g.,* UKFR, a healthcare system reporting few acute COVID-19 patients with CNS or PNS manifestations). When constructing the main Figure 2, we summed the total counts of NNC, CNS, and PNS patients from the number of male and female counts by convention, as these individual counts were less likely required to be obfuscated. In the case of UKFR, the total numbers of CNS and PNS patients were so few that the individual counts of male and female patients with CNS and PNS manifestation were obfuscated to zero, as reflected by the total counts of CNS and PNS patients at UKFR as well as by the outer and middle ring in the gender proportion diagram (*i.e.*, “Male” column). For the gender proportion diagram, we depicted the CNS and PNS ring in a lighter shade of gray to represent the inability to calculate the proportion of male versus female patients stratified by CNS or PNS status due to obfuscation. In contrast, the severity, survival, and readmission diagrams show the proportion of CNS patients in the middle ring because the total number of CNS patients who were non-severe, alive, or readmitted were above the UKFR obfuscation threshold. The counts of PNS patients in these diagrams remain obfuscated.

Importantly, the Cox-proportional hazards models and covariate-adjusted survival curves were performed on the original source data of each healthcare system *without* obfuscation. In the meta-analysis (Fig 6 in the main text), the estimated number of patients at risk (at 0, 30, 60, and 90 days) for clinical events would be insignificantly affected by obfuscation. Given the large size of the overall study population, obfuscation at some of the participating healthcare systems would have minimal, if any, impact on the total patient counts used to evaluate baseline and clinical characteristics or pre-existing health conditions among neurological strata or the survival analyses.

1. Brat GA, Weber GM, Gehlenborg N, Avillach P, Palmer NP, Chiovato L, et al. International electronic health record-derived COVID-19 clinical course profiles: the 4CE consortium. npj Digital Medicine. 2020;3. doi:10.1038/s41746-020-00308-0
